# Unraveling the complexities: morpho-physiological and proteomic responses of pearl millet (*Pennisetum glaucum*) to dual drought and salt stress

**DOI:** 10.3389/fpls.2025.1495562

**Published:** 2025-04-17

**Authors:** Charlie Sithole, Rotondwa Rabelani Sinthumule, Joseph Lesibe Gaorongwe, Oziniel Ruzvidzo, Tshegofatso Bridget Dikobe

**Affiliations:** Unit for Environmental Sciences and Management, Department of Botany, North-West University, Mmabatho, South Africa

**Keywords:** drought stress, morpho-physiological responses, pearl millet, proteomic responses, salt stress

## Abstract

Agriculture is crucial for sustaining the world’s growing population, however various abiotic and biotic stressors, such as drought and salt, significantly impact crop yields. Pearl millet, a nutrient-rich and drought-tolerant crop, is essential as a food source in arid regions. Understanding its response mechanisms to drought and salt stress is important for devising strategies for improved crop performance under water deficit and saline environments. This study investigated the pearl millet’s morphological, physiological, and molecular responses subjected to individual and combined drought and salt stresses for 25 days. Significant reductions in morphological traits, such as plant height, shoot and root fresh weights and lengths, and leaf numbers were observed. Furthermore, key physiological parameters, including chlorophyll content, stomatal conductance, photosynthesis, and transpiration rates notably declined, indicating a complex interaction between stress factors and water regulation mechanisms. Protein expression analysis showed differential upregulation and downregulation patterns between the control and stressed pearl millet plants. Gene ontology mapping identified key biological processes, molecular functions, and cellular components of differentially expressed proteins associated with individual and combined stresses. Notably, a high number of unclassified proteins were identified, indicating the presence of potentially novel proteins involved in stress adaptation. Catalytic and binding activities were the predominant molecular functions detected across treatments suggesting their central role in stress response. These highlighted potential mechanisms of tolerance and adaptation in pearl millet. Overall, this study provides a comprehensive understanding of the detrimental effects of drought and salinity on pearl millet at the morphological, physiological, and proteomic levels, uncovering previously unexplored proteomic responses. These insights offer valuable molecular marker targets for breeding programs aimed at enhancing stress tolerance in pearl millet and related crops.

## 1 Introduction

*Pennisetum glaucum* (L) R. Br, commonly known as pearl millet, is one of the most widely cultivated cereals globally. It is ranked as the sixth most cultivated crop following sorghum, rice, wheat, maize, and barley (Satyavathi et al., 2021). This C_4_ forage crop, a member of the Poaceae family, Paniceae subfamily, is mostly cultivated in semi-arid, drought-prone areas of Africa and the Indian subcontinent, primarily for food purposes (Krishnan and Meera, 2018). It is well known for its efficient production of dry matter, robust photosynthetic capability, and important physiological traits, such as heat tolerance, drought and salt resistance, and survival in low nutrient-content soil compared to other cereal crops like maize and sorghum (Dias-Martins et al., 2018; Toderich et al., 2018). Pearl millet is cultivated globally, covering an approximate area of 30 million hectares across 30 countries, mainly in Africa and Asia (Yadav and Rai, 2013). India has been reported to have the largest cultivation area, ranging from 6.93 million hectares, and the highest production, of up to 8.61 million tons (Directorate of Millets Development, 2020). Its seeds are employed in the poultry industry and consumed by humans (Toderich et al., 2018). However, in economically disadvantaged countries, it is used for food consumption, while its recent uses have expanded to include feed production, alcohol production, food processing, and other industrial purposes (Basavaraj et al., 2010). Furthermore, pearl millet contains higher levels of antioxidants, specifically phenolic compounds which possess anticancer properties (Pandian et al., 2020). Millets, including pearl millet, outperform rice and wheat in their mineral content considered as “high energy” cereals due to their higher oil and protein content, superior amino acid balance, and relatively high vitamin A concentration offering higher nutritional value (Krishnan and Meera, 2018; Malik, 2015). The crop’s adaptability to environmental stress is notable, with superior heat tolerance and efficient soil moisture content, making it an important crop in regions with challenging growing conditions (Khairwal et al., 2007). Additionally, pearl millet is highly recognized for its drought tolerance ability, thriving in regions with annual rainfall as low as 200–600 mm, especially in arid environments such as the Sahel region of West Africa (Burgarella et al., 2018; Ji et al., 2021). It can maintain relatively high leaf water potential under drought conditions, triggering the accumulation of abscisic acid (ABA) to regulate stomatal closure and minimize water loss (Xue et al., 2021). Additionally, *P. glaucum* exhibits deep and extensive root systems, allowing access to water in lower soil horizons, which supports sustained physiological function during prolonged drought (Kholová et al., 2010; Shrestha et al., 2023). Even though the lethal leaf (LA) water potential (Ψ_leaf) of pearl millet is not widely documented, limited research indicates a relationship between xylem water potential and transpiration rates during soil drying, highlighting its ability to regulate water status under drought stress (Cai et al., 2020). Rajasekar et al. (2023) further reported that osmotic stress at -7.5 bar plays a crucial role in determining millet’s resistance to drought.

The majority of crops are affected by drought and salinity, resulting in a significant reduction in crop yield and posing a serious threat to food security. Abiotic stresses such as salinity and drought are particularly detrimental to plant development and growth since they reduce crop yield and are particularly pronounced in arid and semi-arid regions (Singh et al., 2015). Drought and salinity impact various plant activities and induce changes in osmotic and water potential in tissues. These changes impact important plant processes, including growth, photosynthesis, metabolism of lipids, and synthesis of proteins (Meloni et al., 2001). Drought, in particular, reduces crop production, affects water and nutrient interactions, induces physiological changes such as reduced leaf size, and stem lengthening, promotes root proliferation, and lowers plant cell division and expansion rates (Meloni et al., 2001). Additionally, drought stress increases the production of abscisic acid (ABA), which lowers stomatal conductance and transpiration rate (Farooq et al., 2012). On the other hand, the risks associated with salt stress in plants include reduction in water potential that causes tissue desiccation, specific ion effects, and nutrient imbalance due to the high absorption of Na^+^ and Cl^-^ ions, and leads to stomatal closure, increased reactive oxygen species (ROS) production, and oxidative damage to cellular components (Karimi et al., 2005; Rasool et al., 2013). Moreover, increased respiration rates, deteriorated plant development, and diminished photosynthetic efficiency are common manifestations of both salt and drought stresses (Sudhir and Murthy, 2004).

Collectively, drought and salinity form a network of abiotic stress factors that primarily affect pearl millet production globally, which is accelerated by high temperatures and poor soil nutrient status in cultivation areas (Yadav et al., 2012; 2017). Despite pearl millet’s robust and highly adaptive nature to poor environmental conditions than common cereals like maize, rice, and wheat (Varshney et al., 2017). This forage crop is under serious pressure of loss in the future due to climate change complexities. Thus, understanding plant behavior in water deficit and saline conditions is highly important for agricultural sustainability. Given the rising occurrences of drought and salt stress, plants have developed intricate ways to adapt to these harsh environmental conditions. During water scarcity, plants such as pearl millet demonstrate various adaptation mechanisms at the morphological, physiological, biochemical, cellular, and molecular levels. According to Fang and Xiong (2015), these response mechanisms are classified into four basic types of drought resistance: drought avoidance (DA), drought tolerance (DT), drought escape (DE), and drought recovery (DR). Specifically, pearl millet demonstrates drought resilience through three adaptation mechanisms, including DE, DA and DT (Cruz de Carvalho, 2008; Yadav and Sharma, 2016). Shivhare et al. (2020), have demonstrated that drought-tolerant pearl millet genotypes exhibited differentially expressed genes (DEGs) linked to stress-associated phytohormones such as jasmonic acid, ABA, ethylene, salicylic acid and gibberellic acid, as well as secondary metabolite pathways like phenols, wax biosynthesis, mevalonate, shikimate, flavonoids, and alkaloids. In the case of salinity, pearl millet salinity tolerant genotypes responded by its robust antioxidant system, which scavenges ROS, and increased enzyme activity of catalase (CAT) enhancing H_2_O_2_ scavenging, while peroxidases (POX) were involved in the breakdown of peroxide radicals (Jha et al., 2022b).

Understanding plant responses to abiotic stresses such as drought and salt stress is crucial to agricultural sustainability (Ngara and Ndimba, 2014). Proteomics provides comprehensive insights into the molecular mechanisms underlying these responses, thereby contributing to the development of stress-tolerant crops (Kosová et al., 2018). Transcript abundance and genome sequencing projects alone cannot provide insight into the post-translational modifications (PTMs) critical to plants’ growth and development and/or response to different stresses. Thus, functional genomics benefits from proteomics information as it offers a comprehensive understanding of the process (Eldakak et al., 2013).

In this study, we evaluated the growth, physiological traits, and proteomic profiles of abiotic stress-responsive proteins from a drought-tolerant *P. glaucum* (L) R. Br Babala cultivar, leveraging on pearl millet’s drought-tolerant characteristics. Notably, our research addresses the gap in the existing literature by providing a comprehensive physiological and proteomic characterization of dual drought and salt stress-responsive proteins from this cultivar. The findings of this study significantly contribute to the plant sciences discipline by shedding light on the impact of water deficit and salt stress on morphological and physiological traits in plants and identifying the expressed proteins during individual and combined stress conditions.

## 2 Materials and methods

### 2.1 Plant Material and growth conditions

The Babala *P. glaucum* seed cultivar used in this study was obtained from Simply African Seed (Johannesburg, South Africa). Four (4) seeds per pot (12 pots in total) were selected for size homogeneity and good quality. Approximately 48 seeds were collected into a 2 ml Eppendorf tube and surface sterilized with 1 ml of 30% commercial bleach and 0.02% Triton X-100, followed by a brief mixing through a vortex mixer (Labnet international, inc. Model # 230V-EU, Edison, New Jersey, USA) for 2 minutes. Subsequently, the sterilization solution was discarded, followed by rinsing three times with 1.5 ml of sterile distilled water. After sterilization, the seeds were sown in 90 × 15 mm Petri dishes (4 seeds per dish) lined with a moistened filter paper for 10 days. Under laboratory conditions, the sown seed plates were incubated in a growth chamber at the following conditions 25°C 8/16 hours night/day respectively under at 10 000 light lux (Lab Companion, model# GC-300TL, Jeio Tech, Korea). On day 11 about four (4) seedling sprouts were transplanted into each of the twelve (12) plastic plant pots, each with dimensions 154 cm in height, 13 cm at the base and 20 cm in diameter. The pots contained a 2:1 (v/v) mixture of sterile organic soil (Garden Master™ potting mix) and vermiculite (Sanscape vermiculite, Cape Town, South Africa). The transplanted sprouts were grown for 35 days under greenhouse conditions at 25°C/30°C, 8/16 hours night/day respectively under natural light conditions, of approximately 74,000 light lux in mid-afternoon on non-cloudy days (Wilson, 1994). The seedlings were maintained for 18 days by irrigating them daily with 100 ml of sterilized tap water before stress induction.

### 2.2 Drought, salt and dual stress treatment

Following the maintenance period, the plants were divided into four (4) groups: (i) control (water only), (ii) drought (iii) salinity and (iv) drought and salinity (3 pots per condition). The *P. glaucum* control plants were irrigated with 150 ml of sterilized tap water only. Plants subjected to drought stress were irrigated with 150 ml of 15% (w/v) polyethylene glycol 8000 (PEG) solution (Joshi and Dhruve, 2015). In contrast, salinity treatment group plants were irrigated with 150 ml of 200 mM (~20 ds/m) NaCl solution (Al-Shoaibi and Al-Sobhi, 2007); then, the last treatment group was irrigated using a total volume of 150 ml comprising a mixture of 15% (w/v) PEG and 200 mM (~20 ds/m) NaCl solution. All plant groups were irrigated with their respective solutions in two (2) day intervals for 25 days to maintain the initial concentrations. To prevent uneven or one-sided solute distribution and ensure uniform stress levels, the prepared solutions were applied gradually and evenly to pots from top to bottom, ensuring that the entire soil profile received equal amounts of the solution. After the treatment, plants were harvested, and relevant morphological and physiological measurements were recorded for all plant groups. The leaf material was also harvested for proteomic analysis. The harvested leaves were rinsed with sterile distilled water and stored at -80°C for further processing.

### 2.3 Evaluation of the morphological parameters

The morphological parameters of concern included plant height, leaf number per plant pot, leaf area, shoot (length and weight) and root (length and weight) were determined on day 26 after treatment. Analysis of the morphology was performed on all the twelve (12) harvested plants obtained from Section 2.2 by measuring the plant height (cm), shoot and total root lengths (cm) using a ruler, physically counting the number of leaves in each plant pot. To facilitate root extraction for total root length measurement, plant pots were submerged in a bucket of water to moisten the soil and minimize root breakage following the method described by Metcalfe et al. (2007). Fresh root and shoot weights (g) were measured from all twelve plants using a weighing balance (KERN, model # PNS 600-3, Balingen, Germany). The Leaf area was calculated by measuring the leaf length and width following Mananze et al. (2018) equation:


Leaf Area (LA)=α×L×W

Where: L = Length; W = Width and α = Weighing factor (0.75)

### 2.4 Evaluation of the physiological parameters

The physiological parameters of the control, drought, salinity and dual-stressed plant lines were measured and recorded. Physiological parameters such as chlorophyll content, stomatal conductance, photosynthesis, and transpiration of the plants were measured. To measure the chlorophyll content, total chlorophyll was calculated according to the protocol described by Senthilkumar et al. (2021). In this process, 1 gram of pearl millet leaf tissue was ground in an 80% acetone solution to extract chlorophyll a and chlorophyll b. The mixture was then filtered and transferred to a 50 ml Falcon tube, which underwent centrifugation at 2800 × g for 5 minutes using a microcentrifuge (Eins-Sci, Model # E-C15-24.2P, Johannesburg, South Africa). Following centrifugation, the supernatant was transferred to a 100 ml Erlenmeyer flask and diluted with additional acetone solution until the total volume reached 100 ml. The absorbances of the supernatants from all plant groups were measured at wavelengths of 643, 645, 663, and 665 nm using a Genesys 30 spectrophotometer (Thermo Fisher Scientific, Madison, USA), with the solvent (acetone) serving as a blank. The net photosynthetic, stomatal conductance and transpiration rates for all plant groups, were measured following the protocol described by Sinthumule et al. (2022). An infrared gas analyzer (IRGA) LCpro-SD (ADC BioScientific, Hertfordshire, UK) was used to measure the three physiological parameters under constant light conditions of 2000 µmol and CO_2_ levels of 2408 vpm. Three leaves from each plant group were carefully enclosed in the leaf chamber for the evaluation. The rate measurements were carried out for 1.5 hours with 10-second intervals for three minutes at an initial time of T_0_, whereby each leaf was enclosed in an assimilation chamber of the LC pro-SD system set to constant ambient (25-27°C) environmental conditions. The resultant measurements were displayed on the device screen indicating the photosynthetic rates (A) (µmol.m^-2^.s^-1^), stomatal conductance (g_s_) (mol.m^-2^.s^-1^) and transpiration rates (E) (µmol.H_2_O.m^-2^.s^-1^).

### 2.5 Total protein extraction and protein concentration determination

Pearl millet leaf protein extracts were prepared by grinding 100 mg of stored leaves into a fine powder using a sterile mortar and pestle. The total protein extraction was carried out using the NucleoSpin^®^ TriPrep kit (Catalog# 740933.50, Macherey-Nagel, Düren, Germany), with three biological replicates of the control and experiments. The powdered leaf material was lysed with 350 µl lysis buffer and 3.5 µl β-mercaptoethanol, followed by mixing using a vortex mixer (Labnet international, inc. Model # 230V-EU, Edison, New Jersey, USA). The mixture was then transferred into a NucleoSpin filter and centrifuged for 1 minute at 11,000 × g using a microcentrifuge (Eins-Sci, Model # E-C15-24.2P, Johannesburg, South Africa). The resultant lysate was transferred into a NucleoSpin RNA/Protein column and centrifuged for 30 seconds at 11,000 × g. The protein precipitator solution was added to the pellets, and the samples were incubated for 3 minutes at 98°C using a digital dry bath (Labnet International, Inc., Model # D1100, New Jersey, USA). After extraction, two sets of dried pellets were stored in separate 1.5 ml Eppendorf tubes in preparation for sodium dodecyl sulphate polyacrylamide gel electrophoresis (SDS-PAGE) and liquid chromatography mass spectrometry (LC-MS) analysis at -20°C. The total soluble protein concentrations from all the pellet sets were measured using a 2000 Nanodrop spectrophotometer (Thermo Scientific Inc., California, USA).

### 2.6 One-dimensional electrophoresis for total soluble proteins

Following the protocol outlined by Lukhele et al. (2020), one-dimensional electrophoresis (1-DE) was carried out. A one-dimensional (1D) sodium dodecyl-polyacrylamide gel electrophoresis (SDS-PAGE) was prepared with a 12% (v/v) running gel and a 4% (v/v) stacking gel. A total of 10 μg of the extracted total leaf protein samples along with 5 μl of unstained protein marker (Catalog# P7704S New England Biolabs Inc, Massachusetts, USA) were electrophoresed at 200 volts until the dye front reached the bottom of the gel. Protein gels were stained for 60 minutes with a Coomassie Brilliant Blue stain, followed by 60 minutes of de-staining with a de-staining solution comprised of the same components found in the staining solution except for the Coomassie blue powder. Afterward, the produced gel was inspected for visible stress-induced proteins. Images were captured with a Chemi DOCTM Imaging system (Bio-Rad Laboratories Inc., California, USA) using the Bio-Image LabTM Software.

### 2.7 Identification of the stress-induced pearl millet proteins using liquid chromatography-tandem mass-spectrometry

A set of the previously stored protein pellets for the control and stressed plant groups (drought, salt, dual (drought and salt)) was used to evaluate differential protein expression in pearl millet under varying stress conditions through liquid chromatography tandem mass-spectrometry (LC-MS-MS). A total of 16 extracted pearl millet protein samples (4 biological replicates) were used for LC-MS/MS proteomic characterization as outlined in Section 2.7.1- 2.7.3.

#### 2.7.1 Protein preparation and in-gel digestion

Protein pellets obtained from above (Section 2.5) were resuspended in 50 mM Tris-HCl pH 8.0 containing 2% SDS. The protein concentration was measured using the Pierce Bicinchoninic assay (Thermo Fisher Scientific), according to the manufacturer’s instructions. The protein samples (20 μg per sample) were reduced with 5 mM Tris (2-carboxyethyl) phosphine and alkylated with 10 mM 2-chloroacetamide at room temperature for 20 minutes. Followed by sample purification using MagReSyn™ HILIC beads (ReSyn Biosciences) as previously described (Nweke et al., 2020; Baichan et al., 2023). On-bead protein digestion was performed using a 1:10, protease: protein, ratio for sequencing-grade trypsin. The resultant peptides were dried and stored at -80°C before LC-MS analysis.

#### 2.7.2 Liquid chromatography-tandem mass-spectrometry data acquisition

Approximately 1 µg of peptides per sample were analyzed using a Dionex Ultimate 3000 RSLC system coupled to a Sciex 5600 TripleTOF mass spectrometer. Injected peptides were inline de-salted using an Acclaim PepMap C18 trap column (75 μm × 2 cm; 2 minutes at 5 μl. min^−1^ using 2% acetonitrile (ACN)/0.2% formic acid (FA)). Trapped peptides were gradient eluted and separated on a Waters Acquity CSH C18 NanoEase column (75 μm × 25 cm, 1.7 µm particle size) at a flow rate of 0.3 µl. min^−1^ with a gradient of 6-40% solvent B over 30 min (A: 0.1% FA; B: 80% ACN/0.1% FA). Sequential window acquisition of all theoretical mass spectra (SWATH), precursor scans were acquired from 400-1100 *m/z* with 50 milliseconds accumulation time and fragment ions were acquired from 200-1800 *m/z* for 48 variable-width precursor windows with 0.5 Da overlap between windows and 20 milliseconds accumulation time per window.

#### 2.7.3 Bioinformatic analysis and functional annotation

The SWATH data was processed using Spectronaut v17 software (Biognosys), with fixed modifications like carbamidomethylation and variable modifications like N-terminal acetylation and methionine oxidation. The *Sorghum bicolor* reference proteome and common contaminating proteins were used as a search database. A q-value ≤ 0.01 cut-off was applied at precursor and protein levels, and quantification was performed at MS1 and MS2 levels. Label-free cross-run normalization was employed, and candidate dysregulated proteins were filtered at a q-value ≤ 0.01 and absolute Log_2_ fold change ≥ 2. Principal component analysis (PCA) was conducted using ClustVis software (Metsalu and Vilo, 2015) to illustrate the grouping of various treatments in comparison to one another. Proteins were functionally annotated using Gene Ontology, and further classified based on subcellular location, molecular function, biological function, cellular components, protein isoelectric points, and molecular weights using UniProtKB, Expasy, PANTHER, and WoLF PSORT databases. Venny 2.1 (https://bioinfogp.cnb.csic.es/tools/venny/index.html) was used to demonstrate the relationships between identified proteins for various treatment conditions with their respective control comparison.

### 2.8 Statistical analysis

The morpho-physiological results were obtained from three (3) biological replicates between the control and experimental plant groups and subjected to a one-way analysis of variance (ANOVA) (Super-Anova, Statsgraphics Version 7, 1993, Statsgraphics Corporation, USA). Proteomic data was collected from 4 biological replicates. In addition, sample means were compared using the Turkey-Kramer test at a 5% significance level.

## 3 Results

### 3.1 Morphological response of pearl millet under drought, salt and dual stress

**Figure 1:**
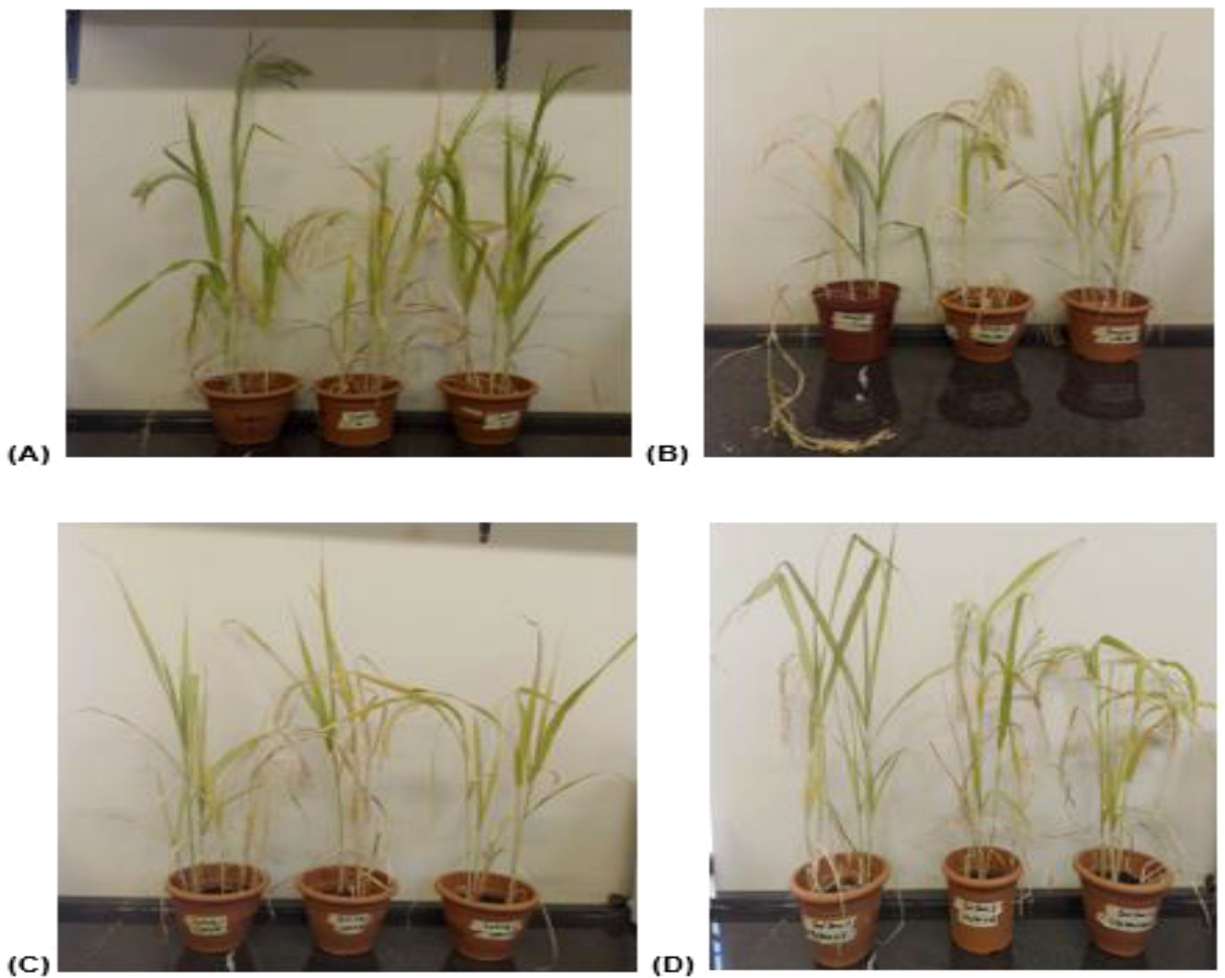
Representations of pearl millet plants after 25 days of experiencing stress. **(A)** indicates non-stressed (control) *P. glaucum* seedlings on day 25 of treatment, **(B)** indicates representatives of drought-stressed *P. glaucum* seedlings on day 25 of treatment, **(C)** indicates representatives of salt-stressed *P. glaucum* seedlings on day 25 of treatment, and lastly **(D)** displays the representatives of dual stressed seedlings at the same treatment period.

**Figure 2:**
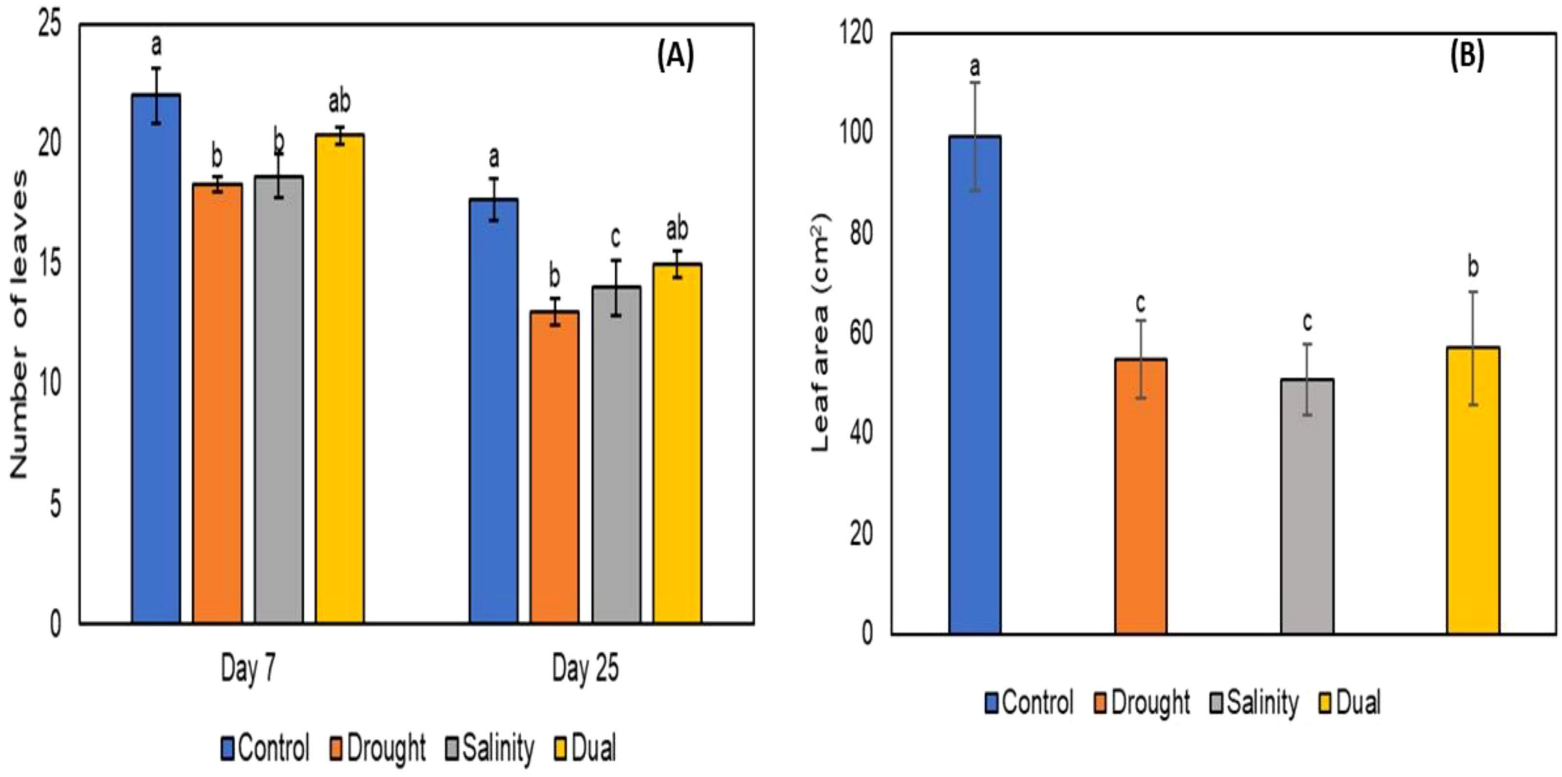
The effect of drought, salt and dual stress on **(A)** Number of leaves per pot on days 7 and 25 of treatment and **(B)** Leaf area. All error bars indicate the standard errors of the means (SEM) of three independent treatments. Lowercase letters refer to the statistical analysis performed using one-way analysis of variance (ANOVA) with the Turkey test at 5% significance and P<0.05 or P<0.01.

**Figure 3:**
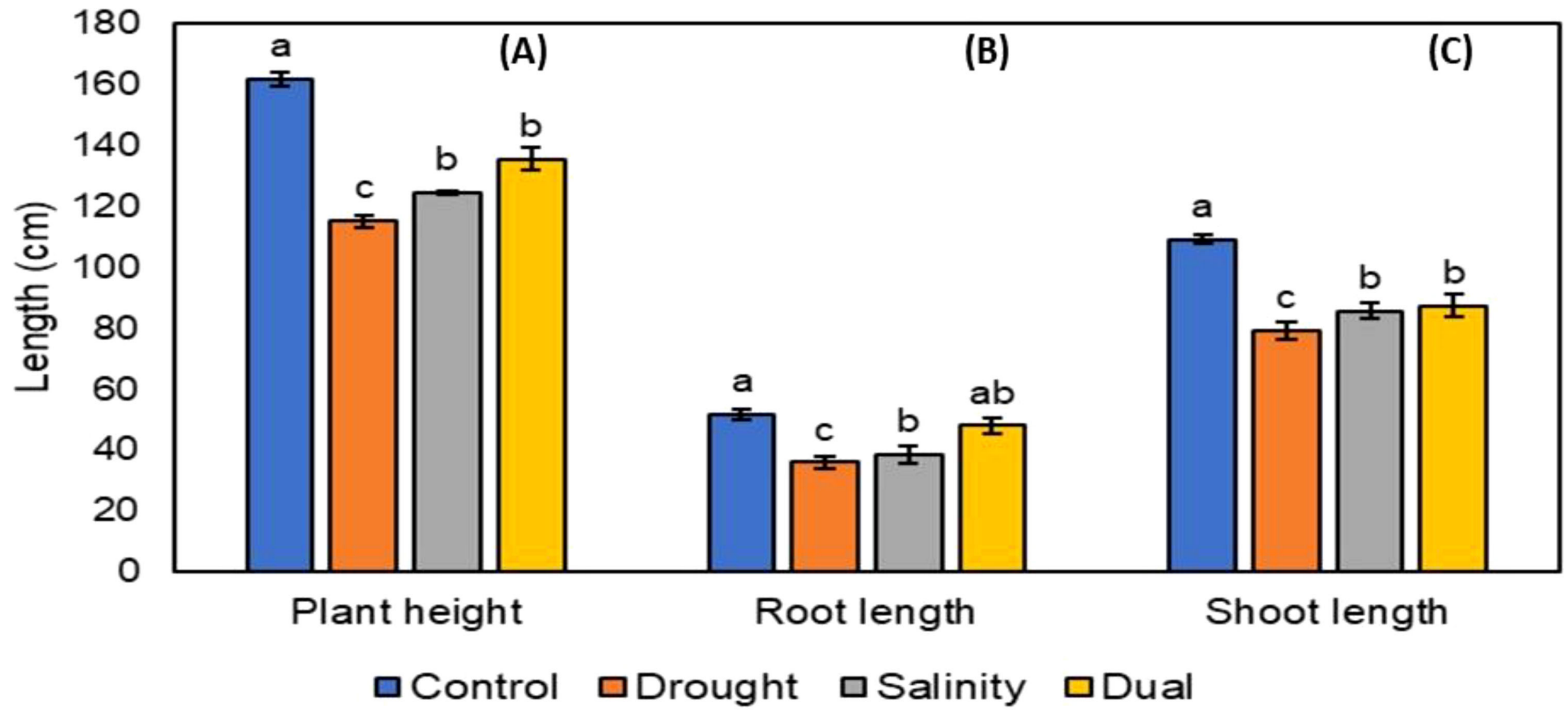
The effect of drought, salt and dual stress on **(A)** Plant height; **(B)** Root length and **(C)** Shoot length. All the data was recorded after 25 days of treatment, where all error bars indicate the standard errors of the means (SEM) of three independent treatments. Lowercase letters refer to the statistical analysis performed using one-way analysis of variance (ANOVA) with the Turkey test at 5% significance and P<0.05 or P<0.01.

**Figure 4:**
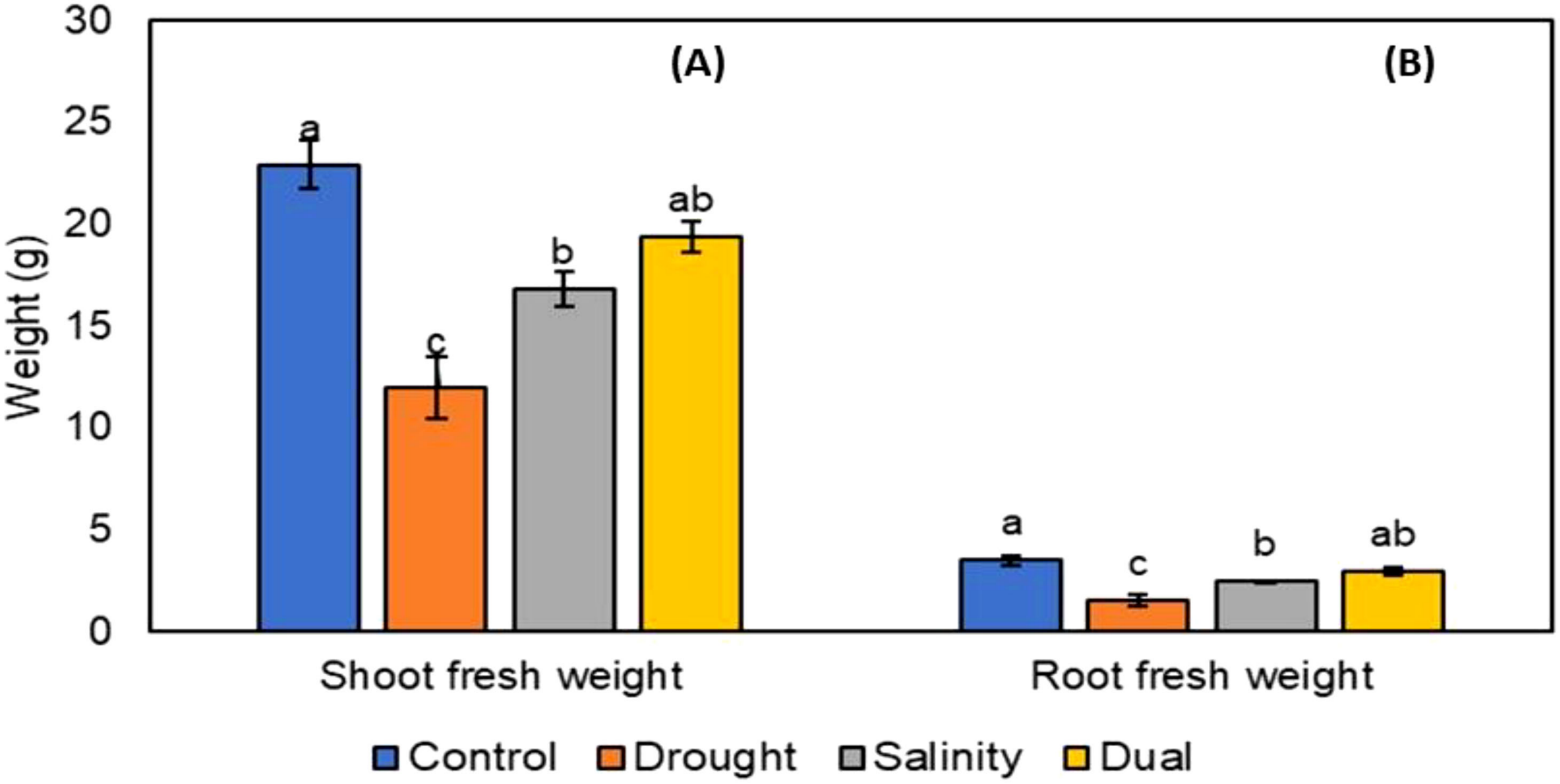
The effect of drought, salt and dual stress on **(A)** Shoot fresh weight and **(B)** Root fresh weight after 25 days of treatment. All error bars indicate the standard errors of the means (SEM) of three independent treatments. Lowercase letters refer to the statistical analysis performed using one-way analysis of variance (ANOVA) with the Turkey test at 5% significance and P<0.05 or P<0.01.

After the successful growth of *P. glaucum* (Babala) cultivar, morphological changes for the control and treated plants (i.e., drought, salinity and dual stress) were observed and documented. Morphological changes in both control and treated plants were captured after 25 days as presented in **Figure 1**. Various phenotypic parameters including plant height, leaf area, leaf numbers per pot, shoot length and weight and lastly root length and weight for the control and treated pearl millet plants were recorded after 25 days of stress induction and their responses are represented in **Figures 2**–**4**. The number of leaves per plant was significantly reduced, with the control group demonstrating a greater number of leaves as compared to plants that experienced drought, salinity and dual stress (p< 0.05) (**Figure 2A**). Specifically, among the treatments, drought was the most affected with reduced leaf number on days 7 and 25 compared to the salinity and dual stressed plants (**Figure 2A**). The leaf area of all the treated plants (i.e., drought, salinity and dual treated) was significantly reduced (p< 0.05) when compared to the control plants which indicated a higher leaf area (**Figure 2B**). In the case of plant height, shoot and root lengths, there was a notable reduction in all parameters under drought, salt and dual stress compared to untreated plants (**Figures 3A–C**). In addition, a significant reduction in shoot and root fresh weights (p< 0.05) was observed in the stressed seedlings (drought, salinity and dual) in comparison to the non-stressed (control) (**Figures 4A** and **3B**). Overall drought demonstrated the most significant impact compared to other treatment conditions (salinity, dual stress), particularly on root and shoot weights.

### 3.2 Physiological response of pearl millet under drought, salt and dual stress

#### 3.2.1 Evaluation of the effect of drought, salt and dual stress on the chlorophyll content

**Figure 5:**
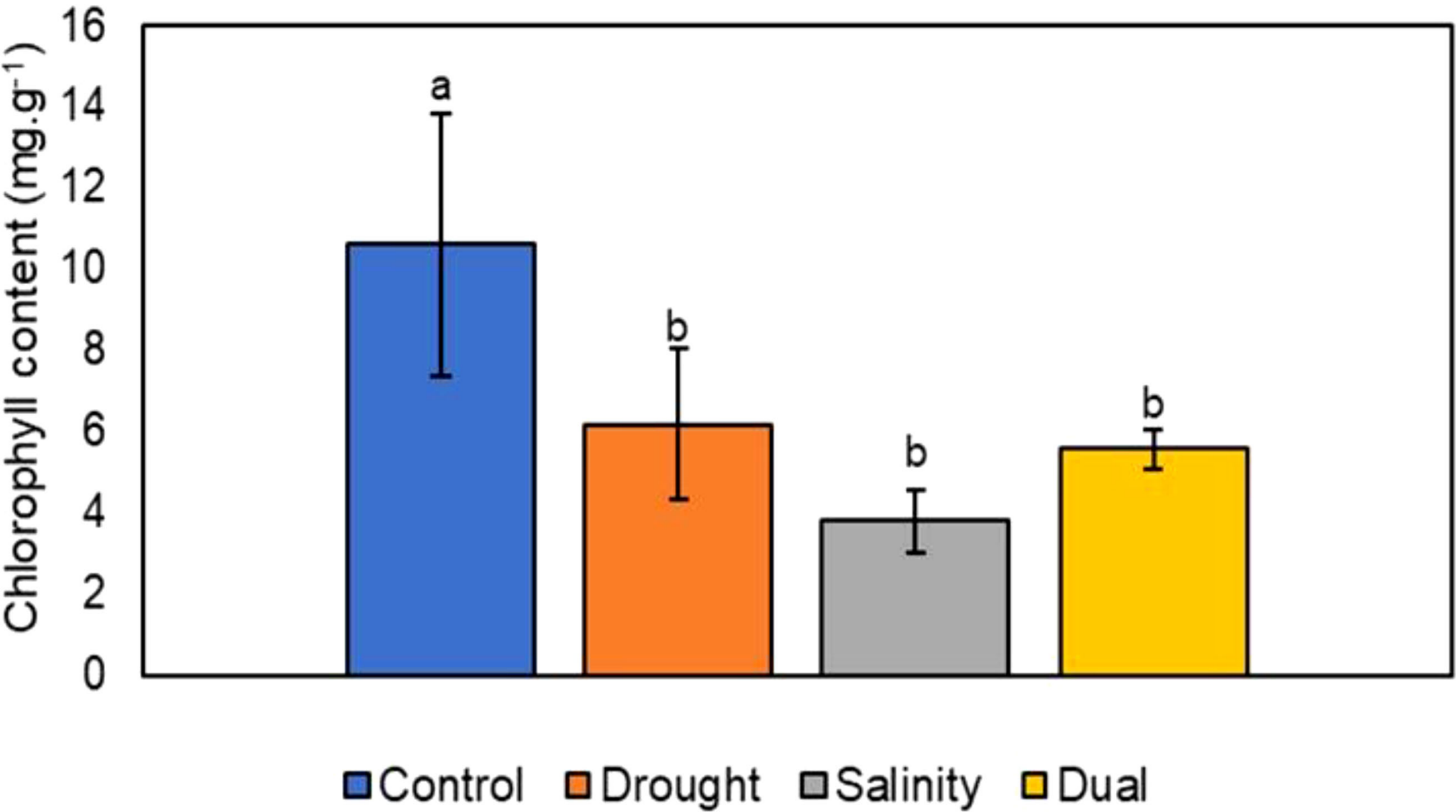
The effects of drought, salinity and dual stress on the chlorophyll content of *P. glaucum* seedlings. Error bars indicate the standard errors of the means (SEM) of three independent treatments. Lowercase letters refer to the statistical analysis performed using one-way analysis of variance (ANOVA) with the Turkey test at 5% significance and P<0.05 or P<0.01.

The effects of drought, salinity and dual stress on the chlorophyll content of *P. glaucum* leaves were assessed after the treatment period. Chlorophyll content of the drought and salt-stressed plants showed a significant (p< 0.05) decrease compared to the control plants. In contrast, a non-significant (p > 0.05) chlorophyll content decrease was demonstrated for the dual treatment compared to the control (**Figure 5**). Salt-stressed plants exhibited a notable reduction compared to the control seedlings and the other two stressed plants (i.e., drought and dual). Notably, among all the treatment groups, salt-stressed plants displayed a major decline in total chlorophyll content compared to the drought, dual stress and control plants.

#### 3.2.2 Stomatal conductance response of *Pennisetum glaucum* to drought, salt and dual stress

**Figure 6:**
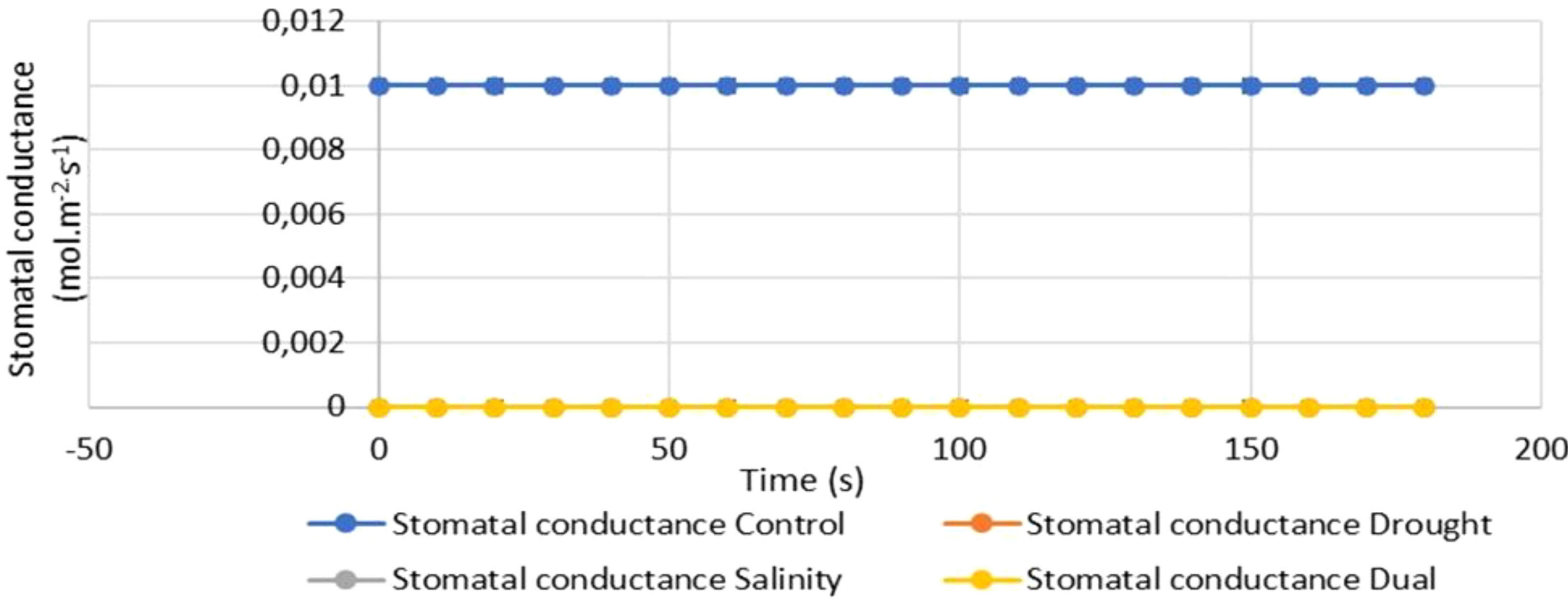
The effects of drought, salinity and dual stress on the stomatal conductance of *P. glaucum* after 25 days of treatment. Error bars indicate the standard errors of the means (SEM) of three independent treatments.

The study further assessed the effects of drought, salinity and dual stress on the stomatal conductance of *P. glaucum* leaves after 25 days of treatment. The stomatal conductance of the stress induced seedlings (drought and salinity) was non-respondent, while the dual stressed seedling was strongly inhibited compared to the non-treated seedlings. Stomatal conductance remained constant across all plant groups at different time intervals (from 0 to 180 seconds). However, it was noted that the stomatal conductance for the control group was slightly higher (0,01 mol.m^-2^. s^-1^) than the dual treated seedlings (0,0 mol.m^-2^. s^-1^) (**Figure 6**).

#### 3.2.3 Photosynthetic response of *Pennisetum glaucum* to drought, salt and dual stress

**Figure 7:**
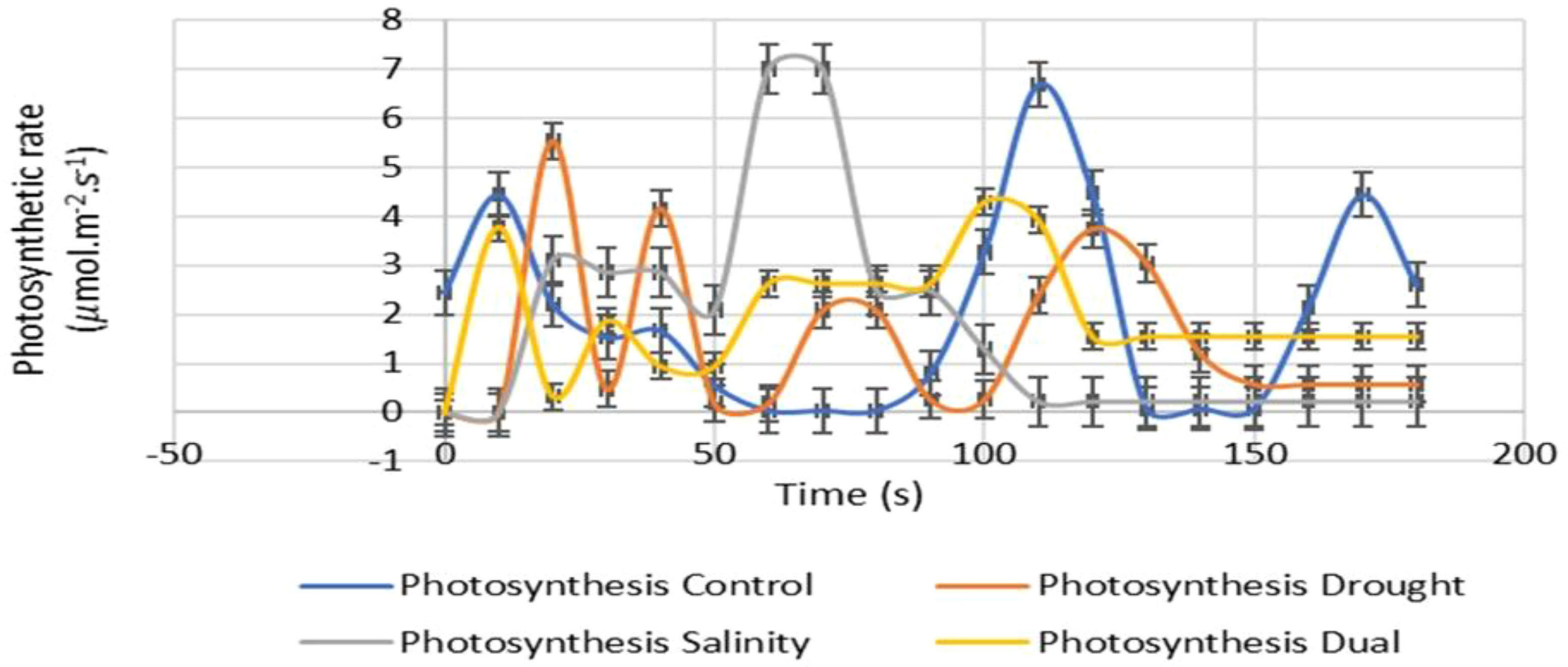
The effects of drought, salinity and dual stress on the photosynthetic rate of pearl millet. The net photosynthesis rates of the control, 15% PEG, 200 mM NaCl and 15% PEG + 200 mM NaCl treated (experimental) leaves. Error bars indicate the standard errors of the means (SEM) of three independent treatments.

The effects of drought, salinity and dual stress on the photosynthetic rates of *P. glaucum* leaves after 25 days of treatment were assessed as shown in **Figure 7**. The experimental seedlings showed lower photosynthesis rates compared to the control, indicating that drought, salinity, and dual stress inhibited photosynthesis. The photosynthetic responses showed a constant rate within the first 10 seconds for both drought and dual-stressed seedlings. However, salinity did not show a consistent rate for the first 10 seconds and its photosynthetic rate for the first 20 seconds was lower than the control. Both drought and dual-stressed plants displayed a rapid increase after the first 10 seconds, followed by a slight increase and unstable photosynthesis rate inhibited by drought and a combination of salt and PEG treatment. The highest photosynthetic rate was observed between 50 and 80 seconds for salt-treated seedlings. At 180 seconds, photosynthetic rate inhibition was noted for the salinity treatment followed by drought and dual stress (**Figure 7**).

#### 3.2.4 Transpiration response of *Pennisetum glaucum* to drought, salt and dual stress

**Figure 8:**
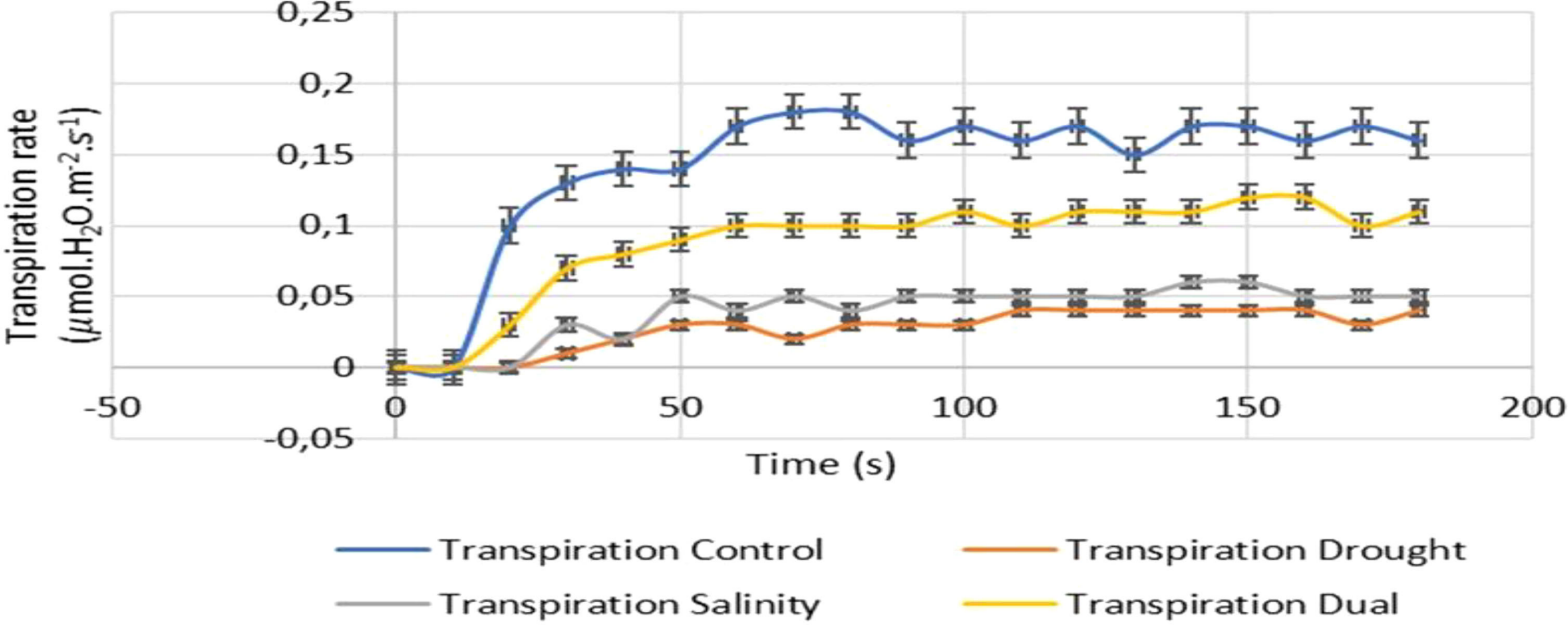
The effects of drought, salinity and dual stress on the transpiration rate of *P. glaucum*. Total transpiration rates following 25 days of treatment for the control, drought, salinity, and dual stressed leaves. Error bars indicate the standard errors of the means (SEM) of three independent treatments.

The transpiration rates were highly affected by induced stresses studied whereby the drought, salinity and dual stressed seedlings were strongly reduced compared to the non-treated seedlings (**Figure 8**). In the first 10 seconds, the transpiration rates of all seedlings had a constant rate, then on the next 10 seconds interval (i.e., 20 seconds), both transpiration rates of the control and dual treated seedlings started to increase, while the other treated seedlings (drought and salinity) remained constant. From the 20 seconds interval, followed by a slight increase and unstable transpiration rate inhibited by drought (PEG) and salt treatment. Both drought and salinity-treated plants showed a slight increase and unstable transpiration rate after the first 20 seconds as inhibited by drought (PEG) and salt treatment. At 180 seconds, the higher transpiration rate inhibition was noted on the drought treatment followed by salinity and dual stress (**Figure 8**).

### 3.3 One-dimensional gel electrophoresis expression profile of pearl millet proteome

**Figure 9:**
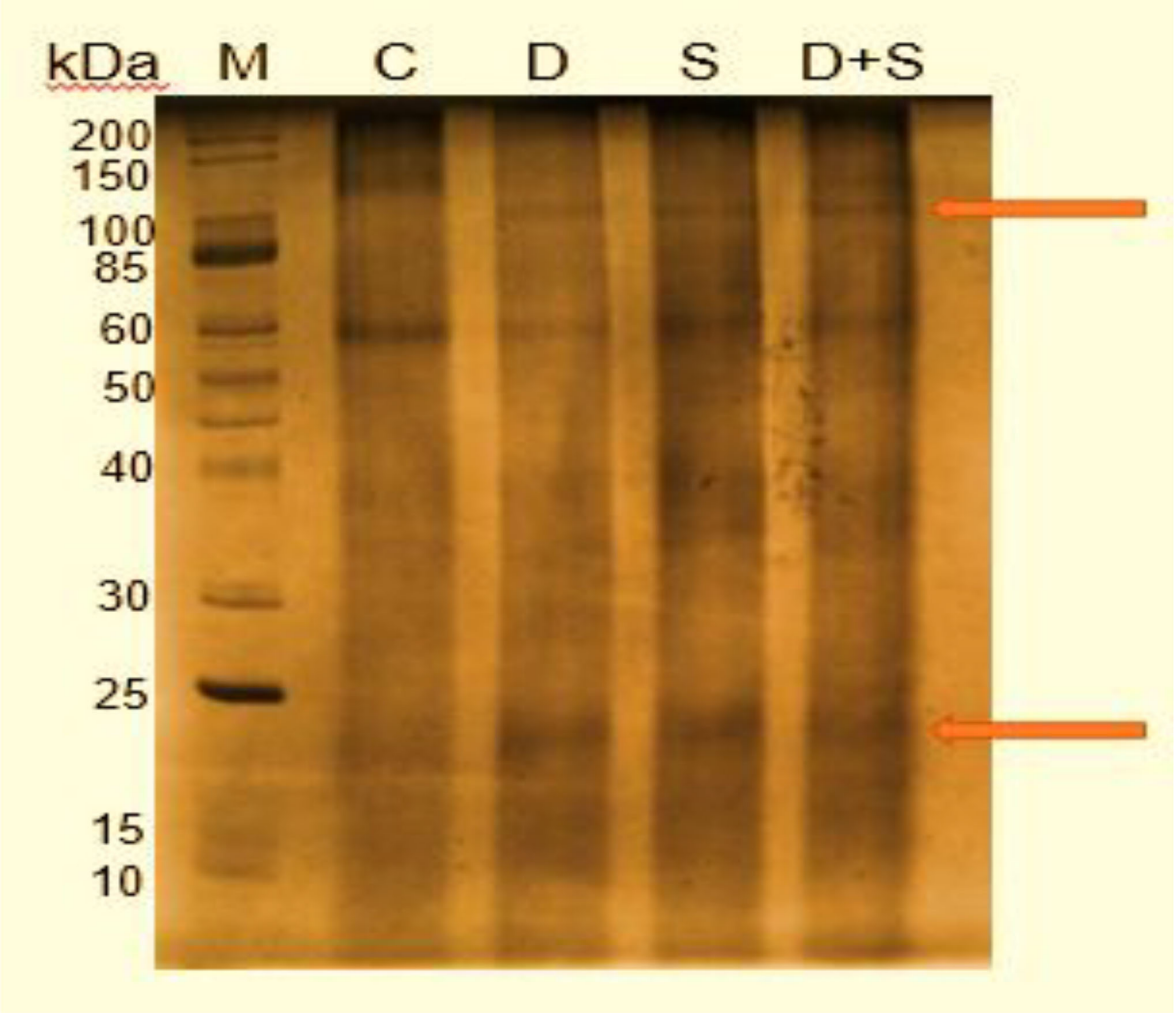
A 12% SDS-PAGE resolution of proteins from the drought, salinity and dual stressed pearl millet seedlings, where lane 1 (M) represents the unstained protein marker (Catalog # P7704S New England Biolabs Inc., Massachusetts., USA); lane 2 (C) represents the control (water treatment only); lane 3 (D) represents the drought experiment (15% PEG treatment); lane 4 (S) represents the salinity experiment (200 mM NaCl treatment); and lane 5 (D+S) represents the dual experiment (15% PEG + 200 mM NaCl treatment).

The quality of extracted proteins from pearl millet leaves was evaluated using one-dimensional SDS-PAGE under different conditions, including drought, salinity, and dual (**Figure 8**). Overexpression was observed for all treatments, with nearly identical banding patterns for both control and experiments. Proteins ranged from 20 to 150 kDa in both conditions, with overexpression at band ranges of 20 and 100 kDa for all treatments. The control showed partial to no expression of these same bands (**Figure 9**).

### 3.4 Identification and classification of drought, salt and dual responsive proteins in pearl millet

#### 3.4.1 Identification of drought, salinity and dual responsive proteins in pearl millet

**Figure 10:**
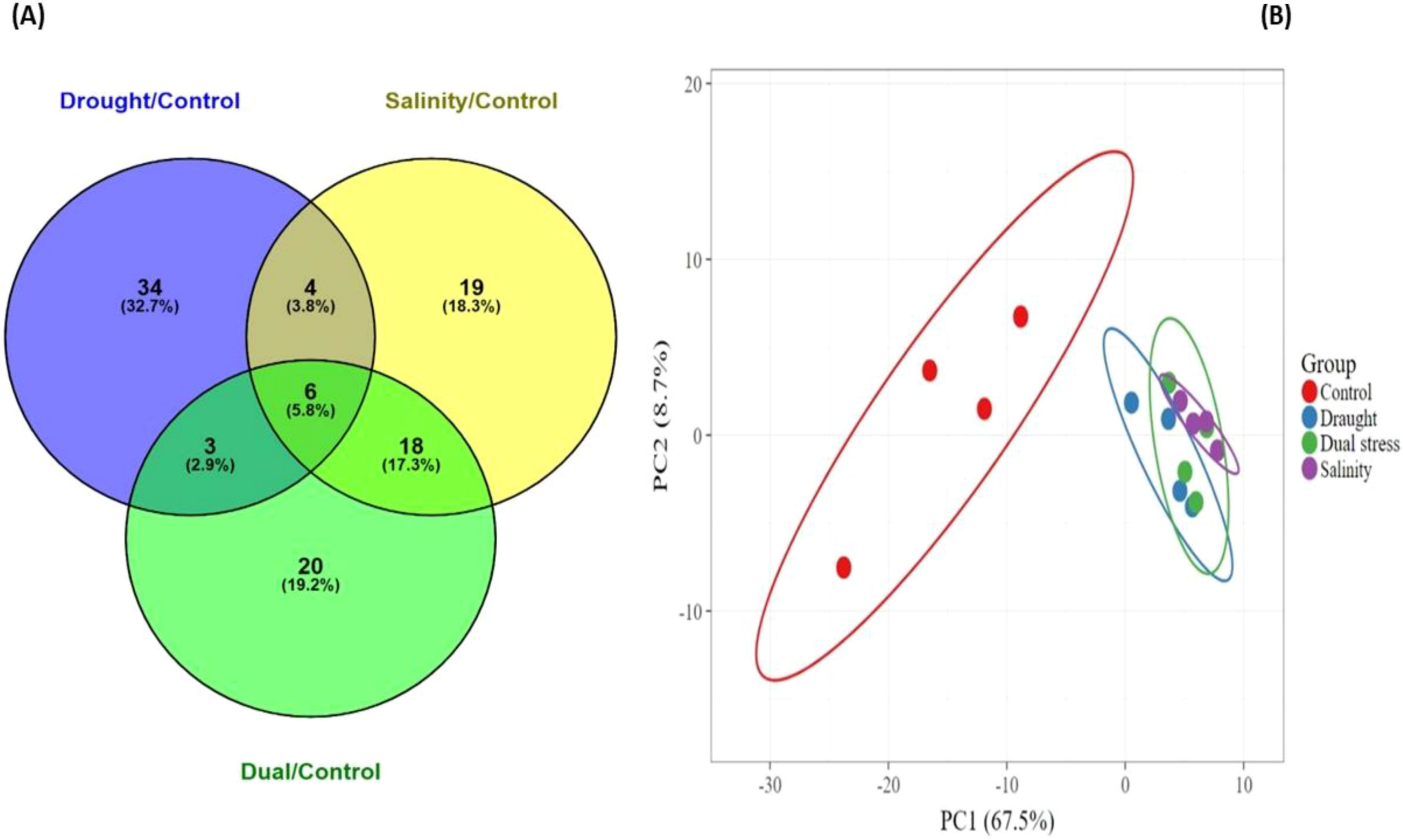
**(A)** Venn diagram demonstrating the relationship between commonly and differentially expressed proteins present in the experiments with their respective comparison control proteins, **(B)** principal component analysis (PCA) of various pearl millet treatment groups: control, drought, salinity, and dual stress (drought + salinity) assessed after 25 days under greenhouse conditions. Principal components 1 and 2 account for 67.5% and 8.7% variability levels respectively.

The study aimed to identify drought, salinity, and dual stress-responsive proteins in pearl millet leaves under varying conditions. Venny 2.1 software was used to compare differentially expressed proteins between experimental conditions and comparison control proteins (**Figure 10A**). Three treatment conditions were selected to identify stress signaling pathways in pearl millet. The protein expression relationship between varying treatment conditions and their unique comparison control proteins was observed. Protein overlap was noted among the various treatments. Under drought, 34 (37.2%) differentially expressed proteins were observed, compared to 20 (19.2%) in the dual with control comparison and 19 (18.3%) in the salinity with control comparison. Additionally, 3 (2.9%) differentially expressed proteins were commonly abundant under drought and dual conditions, while 4 (3.8%) were abundant in both drought and salinity conditions. Under dual and salinity conditions, 18 (17.3%) proteins were commonly abundant. Only 6 (5.8%) of the proteins were common to all conditions with their respective control proteins (**Figure 10A**). The PCA plot further demonstrates distinct differences among the different treatment groups. The control group was highly dispersed along PC1 (67.5%) indicating that the main source of variation results from the stressed plant groups (**Figure 10B**). In contrast, drought, salinity and dual stress groups were closely clustered to one another, suggesting that these treatment groups induced similar molecular responses. PC2 (8.7%) accounts for additional variability in differentiating stress conditions. Notably, the dual stress group shared characteristics with both drought and salinity, and it is found between the two groups. This suggests that pearl millet exhibited overlapping/shared physiological or molecular responses to these stressors. The clear separation of the control group highlights important metabolic or physiological changes under stress conditions, while the tight clustering of the stressed groups reflects shared adaptive mechanisms. Collectively, the PCA suggest that drought and salinity induce comparable responses, with the dual stress demonstrating an intermediate phenotype combining traits from both stressors.

#### 3.4.2 Functional classification of pearl millet proteins under drought, salinity and dual stress

**Figure 11:**
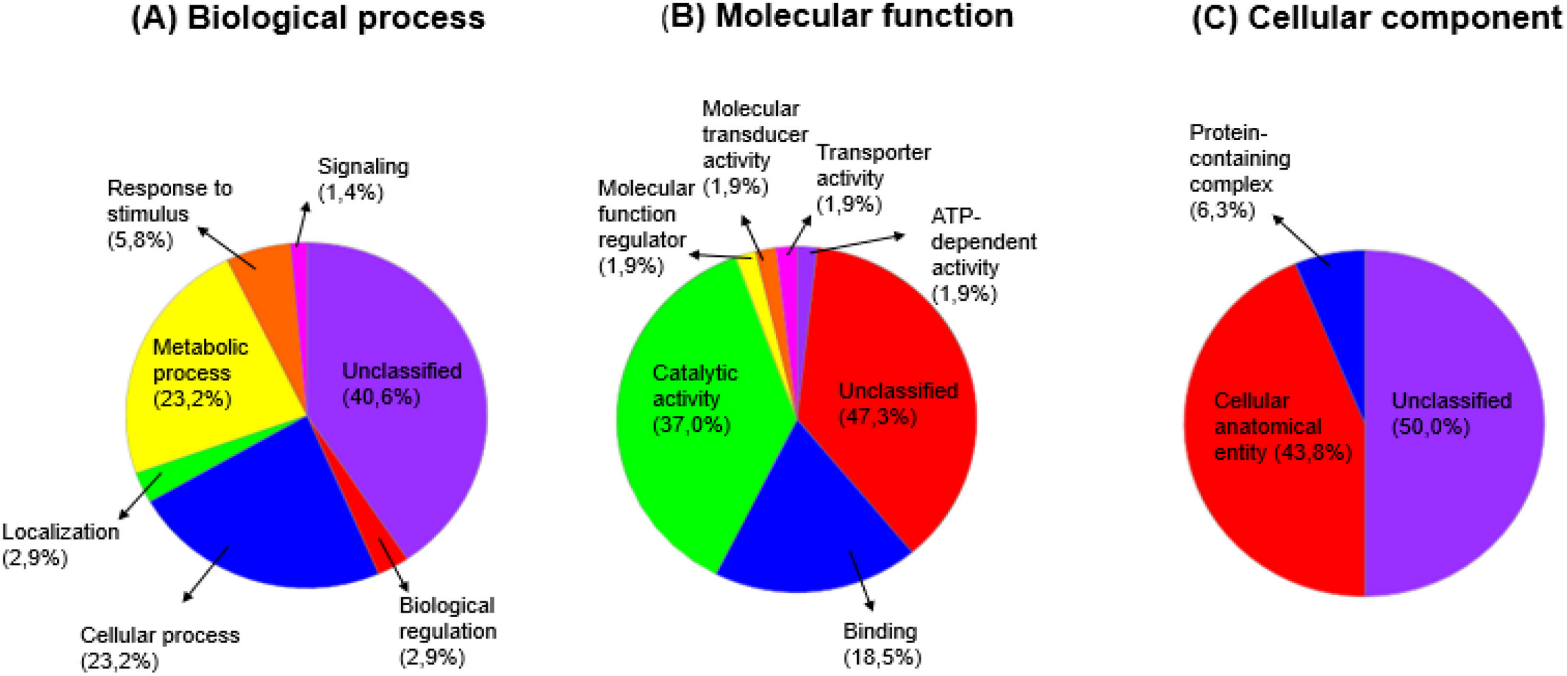
The functional classification of pearl millet proteins under drought (15% PEG) stress with control comparison, whereby: **(A)** Biological process, **(B)** Molecular function and **(C)** Cellular component.

**Figure 12:**
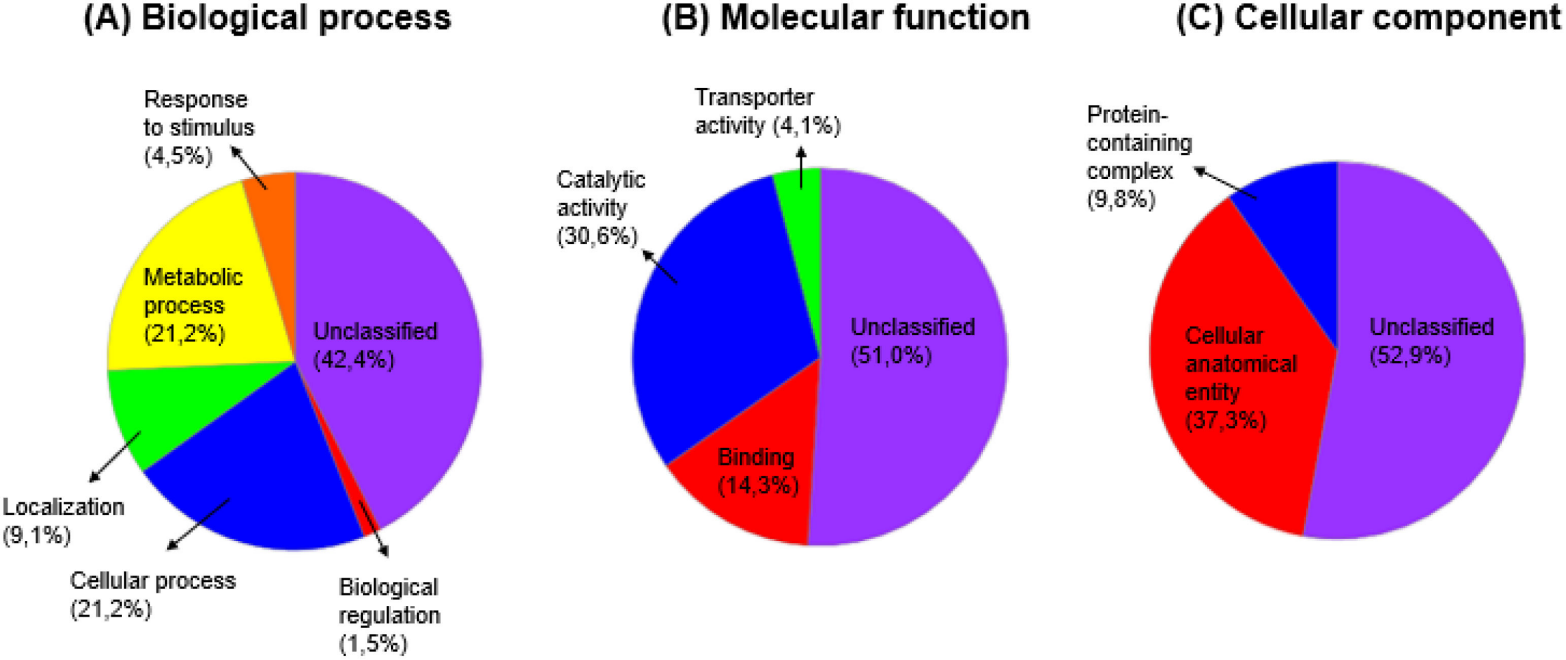
The functional classification of pearl millet proteins under salt (200 mM NaCl) stress with control comparison, whereby: **(A)** Biological process, **(B)** Molecular function and **(C)** Cellular component.

**Figure 13:**
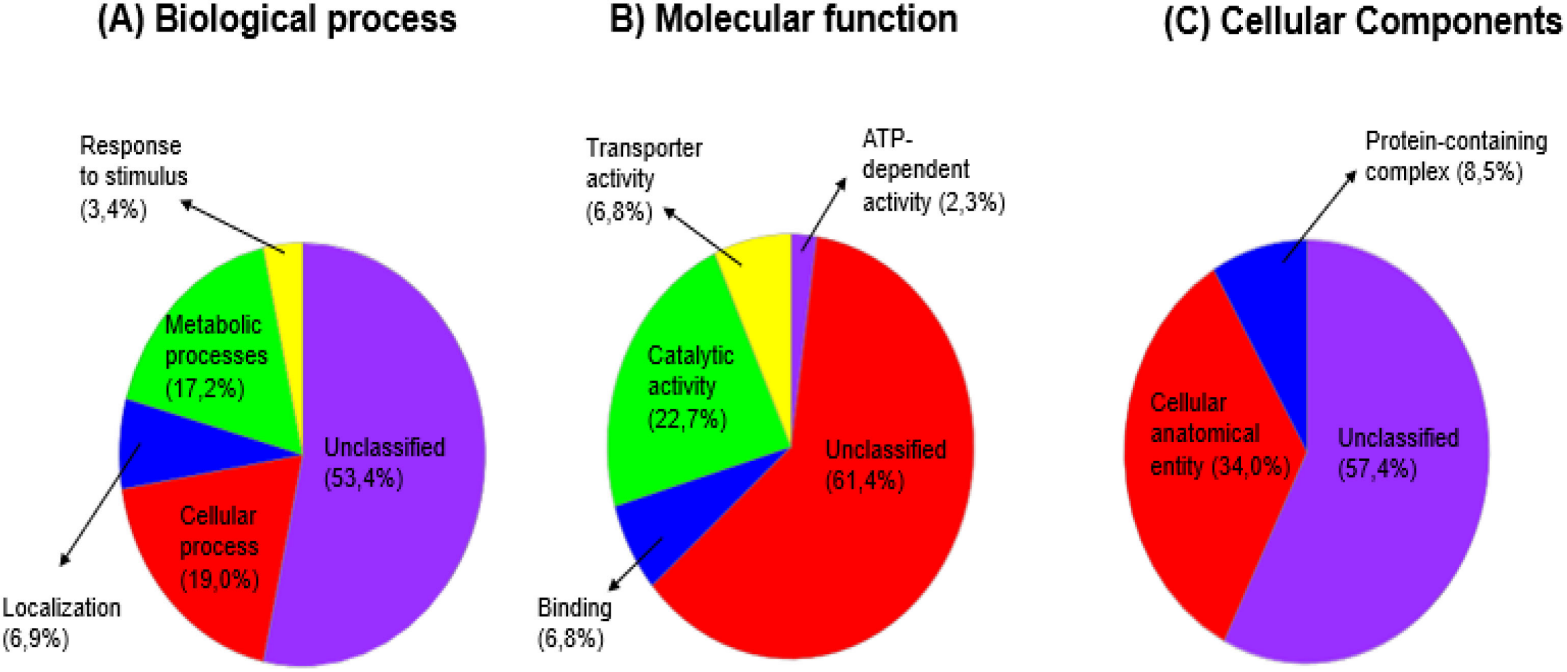
The functional classification of pearl millet proteins under dual (15% PEG + 200 mM NaCl) stress with control comparison, whereby: **(A)** Biological process, **(B)** Molecular function and **(C)** Cellular component.

Panther Gene ontology (GO) was used to determine the biological processes, molecular functions and cellular components of the differentially expressed pearl millet drought, salinity and dual stress responsive leaf proteins each with their control comparison proteins. The results obtained from the GO software are presented in pie charts, illustrating the different protein percentages mapped under various categories. **Figures 11**–**13** display the proportional representation of proteins within specific functional categories. Most differentially expressed leaf proteins were left unclassified. The biological process functional classification revealed that many proteins were unclassified, and majority were involved in cellular and metabolic processes (**Figures 11**–**13**). The essential categories of molecular functional activities that are accountable for the regulation mechanisms of drought, salt, and dual stress, together with their comparative control, are shown in **Figures 11**–**13**. These include catalytic and binding activities, with most activities remaining unclassified. On the cellular component categories, about 85% of proteins were unclassified, followed by 16 - 45% of proteins occupying the cellular anatomical entity. Moreover, the protein containing complex category was between 6 to 10% for all the treatment conditions with their control comparison as shown in **Figures 11**–**13**.

## 4 Discussion

As dry seasons prolong and salinity issues develop in irrigated areas, environmental stresses prevail on farmland threatening crop productivity globally. These stresses result in water scarcity and osmotic effects, that significantly hinder physiological processes and lead to growth reduction (Hu and Schmidhalter, 2005). Throughout their lifespans, crop plants are subjected to various environmental stresses, such as water stress or drought, high or low temperatures, nutrient deficiencies, salt, flooding, and inadequate or excessive light intensity impacting stomatal functioning and the regulation of photosynthesis, respiration, and transpiration, ultimately reducing crop production (Seleiman et al., 2021). Given the diverse ways plants respond to environmental challenges, researchers have invested considerable effort in studying the effects of salinity and drought stress on various plant species. This was to understand the mechanisms used by plants in response to these stresses, with a goal of developing tolerance since plants respond differently to environmental challenges (Satyavathi et al., 2021). Several research studies have been conducted on the effects of combined salinity and drought stress on several crops, including barley (Torun, 2019), cotton (Zhang et al., 2013), wheat (Dugasa et al., 2021), sunflower (Umar et al., 2019), and maize (Sun et al., 2014). On that note, it was therefore necessary in the present study to investigate the morphological, physiological and proteomic characterization of the dual (drought and salt) stress-responsive proteins in *Pennisetum glaucum* (Babala) cultivar. This investigation involved exposing *P. glaucum* seedlings to simulated drought (15% PEG), salinity (200 mM NaCl) and dual stress (15% PEG + 200 mM NaCl) conditions for 25 days under controlled environmental conditions. This study aimed to gain insights into how pearl millet responds to combined stressors (drought and salt) and to identify the proteins involved in this complex response. Such an investigation was crucial for developing strategies to enhance the resilience of crops to multiple environmental stressors, thereby ensuring food security in the face of changing climate patterns.

The study demonstrated significant morphological and physiological alterations in *P. glaucum* seedlings after 25 days of drought, salinity and dual stress exposure. The number of leaves per plant decreased, with control having a greater number of leaves compared to the experimental plants (**Figure 2A**). This reduction in the leaf number and area is consistent with findings from previous studies that support the fact that drought, salinity and dual stress cause decreased leaf growth in crops such as *Pistacia vera* and *Brassica oleracea* (Rahneshan et al., 2018; Sahin et al., 2018). Our results also showed a reduction in leaf area in the experimental plants (drought, salinity and dual stress) compared to control plants (**Figure 2B**). Drought-stressed plants were severely affected, followed by salinity and dual-stressed plants which were moderately affected (**Figure 2B**). These responses align with findings obtained by Omami and Hammes (2006), on *Amaranthus* species that experienced drought and salinity individually and others to dual (drought and salt) stress. Their study hypothesized that a reduction in leaf area could be an avoidance mechanism that allowed plants to minimize the loss of water through transpiration. The ionic balance in plants’ cells according to Wei et al. (2023), becomes disturbed when they experience both drought and salinity. Toxic ions, such as Na^+^, build up as a result of salinity stress and can impede important metabolic and developmental activities. However, because of changed physiological developments meant to handle both stresses at once, when paired with drought, the plant may suffer a less severe initial impact on the leaf area. On the same spectrum, Chowdhury et al. (2016) support the idea that a reduction in a plant’s leaf area is an adaptive mechanism to survive under low-water conditions.

The experimental plants (drought, salinity and dual stressed) displayed a significant decrease in plant height, shoot and root lengths compared to the control plants (**Figures 3A–C**). According to the study results, drought-stressed plants were severely impacted, followed by salinity and lastly dual-stressed plants which were moderately impacted. These results are contrary to the findings obtained by Lu et al. (2021) on *Halegeton glomeratus* species. As plants shift their energy from primary metabolism and biomass accumulation to the activation of stress responses, one of the earliest and most frequent responses to stress is the inhibition of growth (Munns and Tester, 2008). In a study by Yadav et al. (2020), pearl millet cultivars subjected to drought and salinity stress demonstrated a reduction in plant height when compared to non-stressed cultivarsdue to reduced osmotic potential of the soil solution caused by presence of highly soluble salts. In our study combined drought and salt stressed plants, displayed a moderate reduction in plant height when compared to individually stressed plants. Angon et al. (2022) and Mohammadi Alagoz et al. (2023), noted that osmotic stress induced by both drought and salinity negatively impact water uptake. Notably, when plants are faced with combined stress, they may respond less severe than when exposed to individual stress, as they may struggle to distribute resources effectively. This state can result in less drastic declines in plant height due to decreased photosynthesis as indicated by Batool et al. (2020) and Yan et al. (2020). Under dual stress, plants could be struggling to acquire adequate water from the soil, as the presence of salts exacerbates osmotic pressure and induces ionic toxicity complicating the plants’ stress response pathway.

Furthermore, in our study, drought stress imposed a significant reduction on root and shoot lengths of pearl millet cultivar with more severe effects on root length which aligns with the results reported in pea (Okçu et al., 2005) and sorghum (Bibi et al., 2010). It was suggested that the decline in shoot and root lengths may be attributed to a blockage of the xylem and phloem vessels, which prevents the movement of water and minerals (Mohamed and Akladious, 2014). In addition, high soil NaCl concentration could lead to nutrient uptake restriction and physiological water stress, affecting shoot and root lengths (Qaoud et al., 2023). Dual stress having less impact on shoot and root lengths of pearl millet compared to other treatments was also evident in a study conducted by Dugasa et al. (2019), on one of the two genotypes of wheat crops. Their study hypothesized that a larger root system in certain genotypes optimized water absorption by exploring a greater quantity of soil. The latter statement supports our findings on root and shoot length of combined drought and salt stress being moderately impacted, this usually means the plants had larger root system which eventually led to water absorption optimization.

Our study further revealed that drought, salinity and dual stress, significantly affected the overall growth of plants especially the shoot and root fresh weights of pearl millet seedlings (**Figures 4A, B**). Drought-stressed plants were severely impacted, followed by salinity-stressed plants, and dual-stressed plants which were moderately impacted. These findings contradict a study by Ibrahim et al. (2019), which found that plants experiencing dual drought and salinity stress had significantly impacted shoot and root weights higher compared to those experiencing drought and salinity stress individually. Studies by Shivhare and Lata (2019) and Ghatak et al. (2021) have shown that pearl millet genotypes experienced a reduction in shoot and root weights under drought stress. This reduction is attributed to an adaptive response, where plants endure inadequate tissue water content by preserving cell turgor and directing energy to protect seed production. Similar findings were reported by Qaoud et al. (2023), suggesting that the toxic effects of high NaCl concentration may cause a decline. Additionally, Sahin et al. (2018) suggested that elevated matrix and osmotic potentials in soil due to salinity and drought may lead to lower water uptake, resulting in a reduction in shoot and root weights under dual stress conditions. The moderate impact observed on shoot and root weights can be further supported by Angon et al. (2022), who found that antioxidant levels might not increase as significantly under a combination of drought and salt stress compared to when plants are exposed to individual stressor at a time. This observation implies that plants may respond differently to oxidative stress when faced with both salinity and drought concurrently. Such a response explains the reduced overall damage and better maintenance of root and shoot weights despite dual stress conditions.

Furthermore, the analysis of chlorophyll content indicated a reduction in all experimental plants compared to the control. Notably, salinity-treated seedlings were severely affected followed by drought-stressed seedlings while the dual-treated seedlings were least affected (**Figure 5**). This reduction in the chlorophyll content could be attributed to the decreased activity of enzymes that produce chlorophyll in pearl millet (Yadav et al., 2020). Generally, drought stress decreases chlorophyll content in plant leaves at various growth developmental stages (Zafar-ul-Hye et al., 2014), this aligns with our study results. The pronounced effects of salinity on the chlorophyll content of pearl millet may be attributed to the overaccumulation of Na^+^ and Cl^−^ ions, leading to ion toxicity caused by salinity which results in deleterious influence on chlorophyll concentrations (Sharif et al., 2018). While on the other hand, our results showed that combined salinity and drought stress reduced chlorophyll content (**Figure 4**). This decline could be attributed to the toxic effects of salinity that decreased the chlorophyll content in the presence of drought, may be due to chlorophyll photooxidation, overproduction of ROS such as singlet oxygen that destroy chloroplast structure, restriction of chlorophyll biosynthesis, production of chlorophyll degrading enzymes (Bhusal et al., 2019) to Additionally, stomatal conductance, photosynthesis, and transpiration are important plant processes that facilitate certain morphological and biochemical interactive signaling pathways that are necessary for overall plant functioning (Ansari et al., 2019; Qaderi et al., 2019). The physiological rates of these three processes were assessed from randomly selected leaves of all plant groups and recorded at 10-second intervals (**Figures 6**–**8**).

Stomatal conductance rates showed a non-response in the experimental plants (drought, salinity and dual) compared to the control plants. The stomatal conductance decline ranged from 0.01 mol.m^-2^.s^-1^ for the control to 0.00 mol.m^-2^.s^-1^ for all the experimental plants for 180 seconds (**Figure 6**). The non-response in stomatal conductance under drought stress is linked to decreased expression of aquaporin genes and anatomical characteristics, leading to a reduction in chloroplast surface area exposed to intercellular space per unit leaf area (Basu et al., 2016). Furthermore, stomatal conductance was also inactive when pearl millet seedlings were exposed to salt stress compared to non-treated seedlings (**Figure 6**). This non-response was attributed to the deterioration of tissues’ water potential, indicating a decrease in cells’ accessibility to water (Ansari et al., 2019). Water evaporation through the stomata encourages upward water movement and transport of nutrients from the roots to shoots. Reduced water intake from the root zone and reduced CO_2_ availability for photosynthetic use are associated with reduced stomatal conductance. In our study, there was also a reduction in stomatal conductance when comparing dual-stressed seedlings and non-stressed seedlings. According to Xue et al. (2021), for plants to maintain an open stomata, drought lowers the turgor pressure of leaves. Guard cells’ capacity to remain open is compromised as turgor pressure drops, which results in a decrease in stomatal conductance. When combined with salt stress, this effect is enhanced since both stressors cause the plant’s moisture levels to drop. Since there was a reduction in stomatal conductance when our seedlings were exposed to dual stress, similar results were obtained in a study conducted by Siddiqui et al. (2008) in *Brassica napus* genotypes exposed to combined drought and salt stress. The study suggested that salt-tolerant genotypes primarily employ this tactic because stresses like salinity and drought slow down the rate of water uptake by the roots. Another reason could be due to hormone-induced interactions, limiting the rate of photosynthetic activity, CO_2_ assimilation and stomatal opening (Sarker and Oba, 2018).

The data in **Figure 7** reveals that drought, salinity, and their combination (dual stress) adversely affected the photosynthetic rates of experimental plants, which are considerably lower than those of control plants. Drought stress impacts photosynthesis due to both stomatal and non-stomatal constraints, with stomatal closure causing increased photorespiration, reduced nutrient mobility, and leaf senescence, ultimately leading to yield loss (Kumar et al., 2023; Atteya, 2003). Notably, dual-stressed seedlings displayed only a moderate reduction in photosynthesis compared to non-stressed seedlings, and this decrease was less severe than in individually stressed plants. Ma et al. (2020) suggest that this moderation may arise from osmotic adjustments that allow plants to sustain metabolic processes and turgor pressure more effectively under combined stresses. This is further supported by findings from Dugasa et al. (2019), which indicated similar results in wheat genotypes exposed to dual stress, both contributing to increased oxidative stress and the production of reactive oxygen species (ROS) in plant cells (Sabagh et al., 2019).

Notably, an increase in the photosynthetic rate of salinity-stressed plants observed after 50 seconds may be attributed to osmotic adjustment and enhanced ionic homeostasis, which assist with stabilizing photosynthetic machinery under stress (Munns and Tester, 2008). On the other hand, the temporary increase could also result from the activation of antioxidant defense systems that mitigate reactive oxygen (ROS) species, reducing oxidative damage to photosystems (Hasanuzzaman et al., 2021). In contrast, the decrease in photosynthetic rate experienced in the control plants might result from feedback inhibition, whereby accumulated sugars downregulate the photosynthesis process and limit carbon dioxide availability and photoinhibition caused by excess light energy damaging photosystem machinery in the absence of stress-induced protective mechanisms (Taiz et al., 2015).

Similarly, the data presented in **Figure 8** demonstrated that drought, salinity and dual stress had a negative effect on transpiration rates for the experimental plants with lower rates observed compared to the control plants. Stomatal conductance (g_s_) which is a critical factor for regulating transpiration is influenced by stomatal density (Camargo and Marenco, 2011). When plants encounter water stress, they activate signaling pathways that reduce transpiration and leaf growth, with high salt levels in the root zone further exacerbating this decline by causing salt accumulation in the mesophyll and narrowing stomatal openings (Schachtman and Goodger, 2008; da Silva et al., 2008). Our study indicated that dual-stressed seedlings demonstrated a moderate reduction in transpiration rates compared to those subjected to individual stressors. This suggests that while plants typically respond to drought and salinity by closing their stomata to minimize water loss, they may manage gas exchange differently under combined stresses, maintaining some level of photosynthesis despite reduced transpiration (Chaves et al., 2009; Ma et al., 2020). This phenomenon aligns with the findings from Baraldi et al. (2019), which noted similar responses in sweetgum that were subjected to combined drought and salinity.

Additionally, proteomic evaluation was carried out to further understand the effect of drought, salinity and dual stress on *P. glaucum*. The total extracted leaf proteins expressed from all conditions were separated through a 1D SDS-PAGE (**Figure 9**). Protein separation by SDS-PAGE is necessary for separating each protein according to its molecular weight (Gallagher, 2012). Proteins at the range of 20 to 150 kDa in both the control and experiments were highly pronounced while proteins at the range of 10 to 15 KDa were less visible. When evaluating the resultant gel, proteins for control were partially or not expressed compared to the experiments (i.e., drought, salinity and dual) which were overexpressed at an estimated range of 20 to 25 kDa and at 100 kDa due to stress induction. Similar overexpression was observed under stress induction in barley experiencing drought (Hellal et al., 2018) and rice subjected to salinity (Kong-Ngern et al., 2005).

The study further successfully identified differential proteins in pearl millet using liquid chromatography tandem mass spectrometry (LC-MS-MS). All proteins were positively identified under different treatment conditions, including drought, salinity, and dual stress. The study found that 34 proteins changed their abundance uniquely under drought stress, 19 under salinity, and 20 in dual stress compared to their controls (**Figure 10A**). This contradicts previous research by Goussi et al. (2021) on the thylakoid proteomic study of *Eutrema salsugineum* which found that combined drought and salt stress treatment had more identified proteins than individually stress-induced treatments. Interestingly, the lower protein expression and identification observed in our dual stress condition may arise from the complex interactions between drought and salinity. These combined stresses often lead to an increased production of reactive oxygen species (ROS), which can inhibit protein synthesis and disrupt cellular structures. Such disruptions may hinder the expression and identification of functional proteins and lead to selective proteome reorganization, as suggested by Ma et al. (2020). Additionally, principal component analysis (PCA) was conducted to understand the roles of differentially expressed proteins in pearl millet under different treatment conditions (drought, salinity, dual stress). **Figure 10B** demonstrates that the two main clusters have been identified in the PCA. The clustering pattern observed in our PCA plot (**Figure 10B**) is aligned with a previous study on plant abiotic stress response. A separate clustering of the control group observed in this study suggests that stress treatments induced important physiological, biochemical and molecular pathways (Obata and Fernie, 2012). In contrast, drought and salinity treatments demonstrated an overlapping clustering, this may be a result of early response whereby both stresses induce osmotic stress, leading to a reduction in growth, other physiological responses such as stomatal closure, osmoprotectant accumulation, reactive oxygen species (ROS) detoxification reduction and nutrient deficiency (Chaves et al., 2009; Zhu, 2016). Dual stress exhibited intermediate clustering that resembles combined responses to drought and salinity which induce various physiological and biochemical changes as indicated in lettuce (Abdelkader et al., 2024). The current study’s results support the idea that mostly stress-specific adaptations may contribute to common overlapping PCA clustering patterns since plants induce shared response mechanisms such as osmotic adjustment while at the same time regulating unique coping mechanisms against each stress (Munns and Tester, 2008).

The detected proteins were detailed and summarized in Appendices A-C. Most of the sub-cellular locations of the proteins identified were not predicted in all treatments with their respective control comparison. **Appendix A** indicates proteins that were identified under drought stress in comparison to control, **Appendix B** indicates proteins that were identified under salt stress in comparison to control, and lastly, **Appendix C** shows proteins that were identified under dual stress in comparison to control. According to our proteomic analyses, we found that the majority of the proteins identified under combined drought and salt-stressed seedlings **Appendix C** ranged at the isoelectric point (Pi) of 8.0 to 8.99. According to Mohanta et al. (2019), proteins in that Pi range are referred to as basic proteins. Normally osmoregulation process is mediated by basic proteins, which aid plants in adjusting to variations in osmotic pressure induced by salt and drought. Osmotin and other suitable solutes that support cellular structures under stress are examples of these proteins (Athar et al., 2022). Expression of basic proteins may be a sign of changes in metabolic pathways that promote stress-tolerant survival strategies, such as increased production of defense molecules like proline, which aids in osmotic adjustment and maintains the stability of cellular structures (Athar et al., 2022; Yan et al., 2022). In addition, we found that approximately 22 functional proteins that are located in the chloroplast were differentially expressed in salinity (**Appendix B**) and combined stress (**Appendix C**) while very few (i.e approximately 16) functional proteins related to chloroplast were differentially expressed under drought (**Appendix A**). The simultaneous existence of these stresses requires plants to make more extensive adaptations to their photosynthetic apparatus, which leads to the production of proteins involved in energy metabolism and photosynthesis (Goussi et al., 2021; Wen et al., 2022). Our findings revealed that most of the proteins identified were related to chloroplast subcellular components, which are involved in energy metabolism (e.g. starch catabolism or starch biosynthetic processes) and photosynthesis. Furthermore, we found that all proteins identified in seedlings subjected to both combined drought and salinity stress, as well as those under individual salinity stress, are down-regulated as indicated by negative fold change (Appendices B and C). In contrast, among drought-stressed seedlings, approximately 46.81% (22 out of 47 proteins) of the identified proteins are upregulated, as evidenced by positive fold change (**Appendix A**). The upregulation of 46.81% of proteins under drought could be due to active adaptive response, where plants prioritize proteins that mitigate water loss, enhance stomatal closure, and maintain cellular homeostasis where ROS defensive proteins are expressed and various osmoprotectants to cope against drought stress. In contrast, the downregulation of proteins under salinity stress reflects the plant’s metabolic repression due to ionic imbalances caused by osmotic stress and ion toxicity as a strategy to conserve energy and prioritize ionic homeostasis, rather than active protein synthesis. On the other hand, dual stress downregulation response could be due to the synergistic effects of both stresses that intensify stress signals and cause more pronounced repression of metabolic activities, including protein synthesis, as seen in the negative average Log2 ratios for all proteins (Choudhury et al., 2017).

The functional classification of responsive proteins provides clues on the physiological and metabolic pathways that pearl millet use in response to drought, salt and dual stress. The first 47 selected drought, salt and dual responsive proteins each with their control comparison were classified into functional categories using Panther database (**Figures 11**–**13**). Among all the treatment conditions with their respective control comparison, the biological process functional classification revealed that many proteins were unclassified, and the majority were involved in cellular and metabolic processes (**Figures 11**–**13**). Under molecular functional classification, many proteins remained unclassified, followed by proteins that were involved in catalytic and binding activities (**Figures 11**–**13**). Proteins with catalytic and binding activities were abundant in a study conducted by Jha et al. (2022a) when pearl millet cultivars were exposed to salinity. This suggests that antioxidation and transcription factor activities were induced by salt stress, which according to Patel et al. (2020), leads to the synthesis of solutes such as lysine, proline, trehalose, and mannitol. These solutes are primarily used as adaptive mechanisms supporting salinity tolerance. Furthermore, our study highlighted the prevalence of proteins related to the cellular anatomical entity. Cellular entity being at the highest resulted in a study conducted by Kumar et al. (2021) when chickpea salinity tolerant cultivars were induced to salinity. Under biological functional classification, many proteins remained unclassified, followed by the abundance of proteins that were involved in metabolic and cellular processes (**Figures 11**-**13**). Metabolic and cellular processes proteins being abundant in our study concur with the study done by Zhang et al. (2017) when the *Oryza rufipogon* cultivar was exposed to drought stress, this indicated that the cultivar regulated extensive metabolic activity when it was under drought stress.

## 5 Conclusion

In conclusion, the comprehensive investigation of the morphological, physiological, and proteomic profiles of the pearl millet Babala cultivar under drought, salt, and dual stress exposure has provided valuable information into the plant’s response mechanisms. The study effectively elucidated the detrimental effects of these stressors on the treated seedlings and demonstrated the intricate pathways through which pearl millet responds to stress conditions. This study has established correlations between stress conditions and key morphological and physiological traits, such as plant height, shoot and root weights, leaf number, chlorophyll content, stomatal conductance, photosynthesis, and transpiration, it offers a profound understanding of the adaptive strategies employed by pearl millet. Furthermore, the identification of various differentially expressed proteins, despite predominantly unclassified one’s, in the pearl millet leaf proteome analysis sheds light on the potential mechanisms of proteomic tolerance and response to stress. The findings of this study provide a solid foundation for further research and development of strategies to enhance salt, drought, and dual stress tolerance in crop species within the grass family.

## Data Availability

The original contributions presented in the study are included in the article/**Supplementary Material**. Further inquiries can be directed to the corresponding author.
