# Supplementary material for: Unraveling the complexities: morpho-physiological and proteomic responses of pearl millet (Pennisetum glaucum) to dual drought and salt stress
Source: Front Plant Sci. 2025 Apr 17;16:1495562. doi: 10.3389/fpls.2025.1495562 (PMC12044532; doi:10.3389/fpls.2025.1495562)
Supplement: Supplementary file 1 [file Table1.docx]

**APPENDICES**

**Appendix A**

**Table 1:** List of differentially expressed drought (15% PEG) responsive proteins compared to the control identified by LC-MS-MS in *P. glaucum* (Information obtained from UniProtKB, Expasy, and WoLF PSORT databases).

| Accession number | Protein name | Subcellular location | Biological processes | Molecular function | Pi | Mr | Fold change (AVG Log 2 Ratios) |
| --- | --- | --- | --- | --- | --- | --- | --- |
| C5WY75 | Glutathione S-transferase | Cytoplasm, Cytosol | Glutathione metabolic process; Response to chemical | Glutathione transferase activity | 6.14 | 24827.76 | -3.652672346 |
| A0A1B6QD19 | Glycosyl hydrolase family 13 catalytic domain-containing protein | Chloroplast starch grain | Starch catabolic process | Isoamylase activity | 6.28 | 86855.97 | -3.155087225 |
| A0A1W0VRZ3 | FAD/NAD(P)-binding domain-containing protein | None predicted | Aerobic electron transport chain; Phylloquinone biosynthetic process | Hydrolase activity; NAD(P)H dehydrogenase (quinone) activity | 5.59 | 92394.16 | -2.958103886 |
| C5X326 | Peroxidase | Secreted | Hydrogen peroxide catabolic process  ; Response to oxidative stress | Heme binding; Lactoperoxidase activity; Metal ion binding | 5.47 | 34425.15 | -2.84692819 |
| A0A1Z5RCK4 | 1,4-alpha-glucan branching enzyme | Plastid, amyloplast | Glycogen biosynthetic process; Starch biosynthetic process; Starch metabolic process | 1,4-alpha-glucan branching enzyme activity; 1,4-alpha-glucan branching enzyme activity (using a glucosylated glycogenin as primer for glycogen synthesis); Cation binding; Hydrolase activity, hydrolyzing O-glycosyl compounds | 5.30 | 93101.24 | -2.773506538 |
| A0A194YRR3 | Uncharacterized protein | Chloroplast stroma | Beta-carotene catabolic process; Carotene catabolic process | Carotenoid dioxygenase activity; Metal ion binding | 6.26 | 68283.34 | -2.696952111 |
| Q9M6P8 | 1,4-alpha-glucan branching enzyme | Plastid; Amyloplast | Carbohydrate metabolic process; Glycogen biosynthetic process;  Starch biosynthetic process | 1,4-alpha-glucan branching enzyme activity; 1,4-alpha-glucan branching enzyme activity (using a glucosylated glycogenin as primer for glycogen synthesis);  Cation binding; hydrolase activity, hydrolyzing O-glycosyl compounds | 6.52 | 94039.35 | -2.630950526 |
| C5WZE8 | DUF1338 domain-containing protein | None predicted | None predicted | None predicted | 6.57 | 39990.31 | -2.561136675 |
| A0A1Z5RNX3 | Uncharacterized protein | None predicted | None predicted | None predicted | 10.24 | 16202.60 | -2.471822625 |
| A0A1B6PRE5 | Protein kinase domain-containing protein | Chloroplast | Cellular response to nitrogen starvation; Chlorophyll catabolic process; photosynthetic electron transport chain;  Protein phosphorylation; Regulation of anthocyanin biosynthetic process;  Regulation of photosynthesis | ATP binding; protein kinase activity | 5.71 | 76736.05 | -2.440946502 |
| C5YS19 | MPBQ/MBSQ family SAM-binding methyltransferase profile domain-containing protein | Membrane | Methylation | 2-methyl-6-phytyl-1,4-benzoquinone methyltransferase activity | 9.51 | 38637.12 | -2.369818785 |
| C5XC40 | Annexin | Cytoplasm | Response to stress | Calcium ion binding;  Calcium-dependent phospholipid binding | 8.89 | 35781.39 | -2.368789196 |
| A0A1B6Q3A1;C5X0N4 | RRM domain-containing protein; Arginine/serine-rich splicing factor SR32 transcript I | Nuclear speck | mRNA splicing, via spliceosome | RNA binding | 10.07; 10.24 | 29736.72; 31638.80 | -2.352072984 |
| A0A1W0W5Q5 | Enoyl reductase (ER) domain-containing protein | None predicted | None predicted | Metal ion binding;  oxidoreductase activity | 9.33 | 35433.66 | -2.318042618 |
| A4ZVI4 | Starch synthase, chloroplastic/amyloplastic | Plastid, chloroplast; Plastid, amyloplast | Starch biosynthetic process | Glycogen (starch) synthase activity | 6.23 | 66463.62 | -2.30331672 |
| A0A1B6PM68 | ATP-dependent Clp protease proteolytic subunit | Plastid | Protein quality control for misfolded or incompletely synthesized proteins | ATP-dependent peptidase activity;  ATPase binding; Serine-type endopeptidase activity | 6.76 | 31891.31 | -2.284205735 |
| A0A1W0W7T6 | 4-alpha-glucanotransferase | Cytosol | Maltose catabolic process | 4-alpha-glucanotransferase activity;  Beta-maltose 4-alpha-glucanotransferase activity;  Heteropolysaccharide binding;  Starch binding | 5.89 | 108225.76 | -2.243946314 |
| A0A1B6QGF1 | PH domain-containing protein | None predicted | None Predicted | None predicted | 7.68 | 22217.88 | -2.146714053 |
| A0A1B6PH86 | adenylate kinase | Chloroplast; Cytoplasm | Nucleoside triphosphate biosynthetic process;  Phosphorylation | Adenylate kinase activity;  ATP binding;  Cytidylate kinase activity;  Nucleoside diphosphate kinase activity | 8.16 | 65893.74 | -2.108588419 |
| C5YMN8 | Uncharacterized protein | Membrane | Malate transmembrane transport;  Mitochondrial transport;  Oganophosphate ester transport;  Oxaloacetate transport;  Phosphate ion transmembrane transport;  Succinate transmembrane transport;  Sulfate transport; thiosulfate transport | Antiporter activity;  Malate transmembrane transporter activity;  Oxaloacetate transmembrane transporter activity;  Succinate transmembrane transporter activity;  Sulfate transmembrane transporter activity;  Thiosulfate transmembrane transporter activity | 10.21 | 34341.12 | -2.095675128 |
| A0A194YQ83 | L-ascorbate peroxidase | Membrane | Cellular response to oxidative stress;  Hydrogen peroxide catabolic process; Response to reactive oxygen species | Heme binding;  L-ascorbate peroxidase activity;  Metal ion binding; Peroxidase activity | 5.08 | 51232.62 | -2.078808978 |
| C5Y545 | AB hydrolase-1 domain-containing protein | None predicted | None predicted | None predicted | 5.40 | 35726.11 | -2.046581387 |
| C5XQK1 | Uncharacterized protein | None predicted | None predicted | None predicted | 9.54 | 19253.23 | -2.02613463 |
| C5XPV2 | Alpha-1,4 glucan phosphorylase | Cytoplasm | Glycogen catabolic process | Glycogen phosphorylase activity; Linear malto-oligosaccharide phosphorylase activity;  Pyridoxal phosphate binding; SHG alpha-glucan phosphorylase activity | 7.04 | 94479.35 | -2.025225189 |
| C5YAU0 | peptide-methionine (S)-S-oxide reductase | Cytoplasm | Cellular response to oxidative stress | L-methionine-(S)-S-oxide reductase activity;  Peptide-methionine (S)-S-oxide reductase activity | 5.22 | 21032.10 | -2.018159081 |
| C5Z0S1 | Calreticulin | Endoplasmic reticulum lumen | Protein folding;  ubiquitin-dependent ERAD pathway | Calcium ion binding;  carbohydrate binding;  Unfolded protein binding | 6.18 | 46758.20 | +2.147845116 |
| A0A1B6QQ39 | Peptidase A1 domain-containing protein | None predicted | Proteolysis;  Regulation of programmed cell death;  Systemic acquired resistance | Aspartic-type endopeptidase activity | 7.12 | 42488.13 | +2.235630968 |
| C5XK58 | Casein kinase substrate phosphoprotein PP28 domain-containing protein | Cytosol | None predicted | None predicted | 6.90 | 19145.85 | +2.243956677 |
| C5YWY3 | Hyaluronan/mRNA-binding protein domain-containing protein | Cytoplasm;  nucleus | None predicted | RNA binding | 5.74 | 40637.10 | +2.287398079 |
| C5XYI5 | 40S ribosomal protein S24 | Ribonucleoprotein complex;  ribosome | Translation | Structural constituent of ribosome | 10.63 | 15717.80 | +2.453451442 |
| C5Z783 | 4-coumarate--CoA ligase | None predicted | Lignin biosynthetic process;  Response to aluminum ion | 4-coumarate-CoA ligase activity;  CoA-ligase activity;  Trans-cinnamate-CoA ligase activity | 5.13 | 59418.53 | +2.51736737 |
| A0A1W0VYG9 | DUF642 domain-containing protein | None predicted | None predicted | None predicted | 7.15 | 37772.26 | +2.526745104 |
| C5Z0L0 | Dihydrolipoyl dehydrogenase | Mitochondrion | None predicted | Dihydrolipoyl dehydrogenase activity;  Flavin adenine dinucleotide binding | 6.35 | 58749.70 | +2.568511834 |
| C5X978 | C2 domain-containing protein | Cell membrane; nucleus | Abscisic acid-activated signaling pathway | None predicted | 6.51 | 18910.12 | +2.589344411 |
| A0A1B6PTD7 | Carboxypeptidase | None predicted | Proteolysis | Serine-type carboxypeptidase activity | 5.15 | 55959.66 | +6.556690029 |
| C5WRX8 | Sigma 54 modulation/S30EA ribosomal protein C-terminal domain-containing protein | Cytosolic small ribosomal subunit | Negative regulation of translational elongation;  Primary metabolic process | mRNA binding;  Ribosomal small subunit binding;  Ribosome binding | 6.42 | 33639.38 | +2.708559443 |
| C5YG26 | Glutathione transferase | Cytoplasm | Glutathione metabolic process; Response to toxic substance | Glutathione binding;  Glutathione transferase activity | 6.45 | 25613.02 | +2.81132171 |
| C5Z3A0 | SCP domain-containing protein | Extracellular space | Response to biotic stimulus | None predicted | 9.12 | 15392.42 | +2.825642382 |
| Q4VQB2 | Pathogenesis-related protein 10b | Nucleus | Abscisic acid-activated signaling pathway; Defense response; Regulation of protein serine/threonine phosphatase activity;  Response to biotic stimulus | Abscisic acid binding; Protein phosphatase inhibitor activity;  Signaling receptor activity; | 5.34 | 17013.57 | +2.88310884 |
| A0A1B6QP78 | Anthranilate synthase | None predicted | Tryptophan biosynthetic process | Anthranilate synthase activity | 6.85 | 66566.59 | +2.965646623 |
| A0A1Z5R3J8 | Glucan endo-1,3-beta-D-glucosidase | None predicted | Carbohydrate metabolic process | Hydrolase activity, hydrolyzing O-glycosyl compounds | 6.41 | 33548.07 | +3.019517514 |
| C5YCD6 | Phenylalanine ammonia-lyase | Cytoplasm | Cinnamic acid biosynthetic process;  L-phenylalanine catabolic process | Ammonia-lyase activity;  phenylalanine ammonia-lyase activity | 6.25 | 75650.83 | +3.14813074 |
| C5Y1P6 | Apyrase | Membrane | Nucleoside diphosphate catabolic process | ATP binding;  Nucleoside diphosphate phosphatase activity | 4.97 | 46522.28 | +3.193716828 |
| A0A1B6QET0 | Beta-amylase | None predicted | Polysaccharide catabolic process | Amylopectin maltohydrolase activity;  Beta-amylase activity | 4.96 | 55035.22 | +3.200756935 |
| C5X5P1 | Apyrase | Membrane | Nucleoside diphosphate catabolic process | ATP binding;  Nucleoside diphosphate phosphatase activity | 6.42 | 50166.45 | +3.540376923 |
| C5XES7 | Lipoxygenase | None predicted | Fatty acid biosynthetic process; lipid oxidation; Oxylipin biosynthetic process | Metal ion binding; Oxidoreductase activity, acting on single donors with incorporation of molecular oxygen, incorporation of two atoms of oxygen | 5.77 | 98192.88 | +3.545999984 |
| A0A1Z5R6H7 | Translocon-associated protein subunit beta | None predicted | None predicted | None predicted | 7.80 | 19419.11 | +3.557096002 |

**NB:** Fold change values are shown for drought stress where + and − symbols depict up and downregulation of the identified proteins, respectively.

**Appendix B**

**Table 2:** List of differentially expressed salinity responsive proteins compared to the control identified by LC-MS-MS in treated *P. glaucum* (Information obtained from UniProtKB, Expasy, and WoLF PSORT databases).

| Accession number | Protein name | Subcellular location | Biological processes | Molecular function | Pi | Mr | Fold change  (AVG Log 2 Ratios) |
| --- | --- | --- | --- | --- | --- | --- | --- |
| C5X326 | Peroxidase | Secreted | Hydrogen peroxide catabolic process; Response to oxidative stress | Heme binding;  Lactoperoxidase activity;  Metal ion binding | 5.47 | 34425.15 | -4.517152391 |
| C5XBU3 | peptidylprolyl isomerase | None predicted | None predicted | Peptidyl-prolyl cis-trans isomerase activity | 10.06 | 23044.29 | -4.42536385 |
| A0A1Z5RCK4 | 1,4-alpha-glucan branching enzyme | Plastid, amyloplast | Glycogen biosynthetic process; Starch biosynthetic process;  Starch metabolic process | 1,4-alpha-glucan branching enzyme activity; 1,4-alpha-glucan branching enzyme activity (using a glucosylated glycogenin as primer for glycogen synthesis); Cation binding;  Hydrolase activity, hydrolyzing O-glycosyl compounds | 5.30 | 93101.24 | -4.324755504 |
| Q9M6P8 | 1,4-alpha-glucan branching enzyme | Plastid, amyloplast | Carbohydrate metabolic process;  Glycogen biosynthetic process;  Starch biosynthetic process | 1,4-alpha-glucan branching enzyme activity; 1,4-alpha-glucan branching enzyme activity (using a glucosylated glycogenin as primer for glycogen synthesis); cation binding; hydrolase activity, hydrolyzing O-glycosyl compounds | 6.52 | 94039.35 | -3.986126375 |
| C5YV95 | Sugar phosphate transporter domain-containing protein | Plastid, chloroplast menbrane | Monocarboxylic acid transport | Antiporter activity;  Carboxylic acid transmembrane Transporter activity; Organophosphate ester transmembrane transporter activity | 9.86 | 45116.04 | -3.893247931 |
| C5YJS0 | RRM domain-containing protein | Cytoplasm;  Nucleus;  Ribonucleoprotein complex | None predicted | mRNA binding | 9.57 | 28788.87 | -3.70321687 |
| A1E9V4;  C5XL99 | Cytochrome b6; Cytochrome b/b6 N-terminal region profile domain-containing protein | Plastid, chloroplast thylakoid membrane; membrane | Photosynthesis;  Respiratory electron transport chain | Electron transporter, Transferring electrons within cytochrome b6/f complex of photosystem II activity; Metal ion binding;  Electron transfer activity | 8.89; 9.06 | 24164.57; 25946.55 | -3.627335548 |
| C5Y7U2 | Chlorophyll a-b binding protein, chloroplastic | Plastid, chloroplast thylakoid membrane | Photosynthesis, light harvesting in photosystem I; Response to light stimulus | Chlorophyll binding | 5.32 | 30111.56 | -3.62660312 |
| A0A194YU41 | Cytochrome P450 | None predicted | Xanthophyll biosynthetic process | Carotene beta-ring hydroxylase activity; Heme binding;  Iron ion binding; oxidoreductase activity, acting on paired donors, with incorporation or reduction of molecular oxygen | 5.93 | 70015.00 | -3.562293945 |
| A0A194YQ92 | CBM20 domain-containing protein | Chloroplast | Protein autophosphorylation;  starch metabolic process | ATP binding;  carbohydrate kinase activity; Phosphoglucan, water dikinase activity;  Starch binding | 5.55 | 133466.71 | -3.523866544 |
| A1E9Q9 | Photosystem II D2 protein | Plastid, chloroplast thylakoid membrane | Photosynthetic electron transport in photosystem II | Chlorophyll binding; Electron transporter, Transferring electrons within the cyclic electron transport pathway of photosynthesis activity; Iron ion binding;  oxygen evolving activity | 5.34 | 39402.79 | -3.457869021 |
| C5YEE1 | VDE lipocalin domain-containing protein | Membrane | Chlorophyll metabolic process;  Xanthophyll cycle | Violaxanthin de-epoxidase activity | 5.26 | 50602.07 | -3.394897603 |
| C5Y545 | AB hydrolase-1 domain-containing protein | None predicted | None predicted | Hydrolase activity | 5.40 | 35726.11 | -3.378769272 |
| C5XEW1 | Peptidyl-prolyl cis-trans isomerase | None predicted | Protein peptidyl-prolyl isomerization | Peptidyl-prolyl cis-trans isomerase activity | 9.54 | 29798.03 | -3.351837045 |
| A4ZVI4 | Starch synthase, chloroplastic/amyloplastic | Plastid, chloroplast; Plastid, amyloplast | Starch biosynthetic process | Glycogen (starch) synthase activity | 6.23 | 66463.62 | -3.336846852 |
| A0A1B6Q9X0 | NAD-dependent epimerase/dehydratase domain-containing protein | Cytosol | rRNA processing | RNA binding | 9.11 | 45578.23 | -3.323556542 |
| C5Z843 | Cyanobacterial aminoacyl-tRNA synthetase CAAD domain-containing protein | Membrane | None predicted | None predicted | 9.04 | 15911.76 | -3.316124952 |
| A1E9U1 | Cytochrome b559 subunit alpha | Plastid, chloroplast thylakoid membrane | Photosynthetic electron transport chain | Electron transfer activity;  Heme binding;  Iron ion binding | 4.64 | 9438.71 | -3.253506678 |
| A0A1B6P9W0 | Protein kinase domain-containing protein | Chloroplast | Circadian rhythm;  Photosynthetic acclimation;  Protein phosphorylation;  Regulation of photosynthesis, Light reaction | ATP binding;  Protein serine/threonine kinase activity | 9.37 | 62836.82 | -3.248272212 |
| A1E9V0 | Photosystem II CP47 reaction center protein | Plastid, chloroplast thylakoid membrane | Photosynthetic electron transport in photosystem II | Chlorophyll binding; Electron transporter, transferring electrons within the cyclic electron transport pathway of photosynthesis activity | 6.06 | 5607034 | -3.238148333 |
| C5YTC0 | NAD-dependent epimerase/dehydratase domain-containing protein | Cytosol | rRNA processing | RNA binding | 8.88 | 42152.90 | -3.224052474 |
| C5YHK1 | STI1/HOP DP domain-containing protein | None predicted | Protein targeting to chloroplast | Chloroplast targeting sequence binding | 4.48 | 36237.16 | -3.122359166 |
| A0A1W0VRZ3 | FAD/NAD(P)-binding domain-containing protein | None predicted | Aerobic electron transport chain;  Phylloquinone biosynthetic process | Hydrolase activity;  NAD(P)H dehydrogenase (quinone) activity | 5.59 | 92394.16 | -3.120677955 |
| C5YBC8 | Phosphoglycolate phosphatase | None predicted | None predicted | Metal ion binding;  Phosphatase activity | 5.42 | 38496.48 | -3.100764323 |
| C5YY74 | plastoquinol--plastocyanin reductase | Plastid, chloroplast thylakoid membrane | None predicted | 2 iron, 2 sulfur cluster binding;  Electron transporter, transferring electrons within cytochrome b6/f complex of photosystem II activity;  Metal ion binding;  oxidoreductase activity; Plastoquinol--plastocyanin reductase activity | 8.20 | 23971.16 | -3.072388164 |
| C5XEU1 | Rhodanese domain-containing protein | None predicted | Cellular response to calcium ion;  De-etiolation;  regulation of stomatal closure | None predicted | 5.03 | 64361.76 | -3.06234644 |
| C5Z5R1 | fructose-bisphosphate aldolase | cytosol | Fructose 1,6-bisphosphate metabolic process; Glycolytic process | Fructose-bisphosphate aldolase activity | 7.57 | 37985.83 | -3.050838462 |
| A0A194YL80 | Uncharacterized protein | Photosystem II oxygen evolving complex | Photosystem II assembly;  Photosystem II stabilization | Oxygen evolving activity | 5.89 | 34805.81 | -3.048355873 |
| C5XWW2 | Uncharacterized protein | VCP-NPL4-UFD1 AAA ATPase complex | ER-associated misfolded protein catabolic process; Ubiquitin-dependent ERAD pathway | polyubiquitin modification-dependent protein binding | 5.76 | 34591.23 | -3.046265407 |
| C5XJS9 | GTP-binding protein SAR1A | Endoplasmic reticulum; Golgi apparatus | Endoplasmic reticulum to Golgi vesicle-mediated transport; Intracellular protein transport; Membrane organization;  Positive regulation of protein exit from endoplasmic reticulum;  Regulation of COPII vesicle coating;  vesicle organization | GTP binding;  GTPase activity | 6.97 | 22026.44 | -3.037528968 |
| C5Z316 | Pyruvate phosphate dikinase AMP/ATP-binding domain-containing protein | None predicted | Cold acclimation;  Phosphorylation;  Response to symbiotic fungus; Starch catabolic process | Alpha-glucan, water dikinase activity; ATP binding;  mRNA binding | 5.79 | 164162.60 | -3.02760298 |
| C5YMN8 | Uncharacterized protein | Membrane | Malate transmembrane transport; Mitochondrial transport; Organophosphate ester transport;  Oxaloacetate transport;  Phosphate ion transmembrane transport;  succinate transmembrane transport; sulfate transport;  thiosulfate transport | Antiporter activity; malate transmembrane transporter activity;  Oxaloacetate transmembrane transporter activity;  Succinate transmembrane transporter activity;  Sulfate transmembrane transporter activity;  Thiosulfate transmembrane transporter activity | 10.21 | 34341.12 | -3.015776024 |
| A0A1B6PHP7 | Chlorophyll a-b binding protein, chloroplastic | Plastid, chloroplast thylakoid membrane | Photosynthesis, light harvesting in photosystem I;  Response to light stimulus | Chlorophyll binding | 6.59 | 27322.98 | -2.995225191 |
| A1E9S3 | Photosystem I P700 chlorophyll a apoprotein A2 | Plastid, chloroplast thylakoid membrane | Photosynthesis | 4 iron, 4 sulfur cluster binding;  Chlorophyll binding;  Electron transfer activity;  Magnesium ion binding | 6.63 | 82517.87 | -2.965856676 |
| C5Y669 | Uncharacterized protein | None predicted | None predicted | None predicted | 6.13 | 39888.15 | -2.938942377 |
| A1E9Q4 | Photosystem II protein D1 | Plastid, chloroplast thylakoid membrane | Photosynthetic electron transport in Photosystem II; Response to herbicide | Chlorophyll binding; Electron transporter, transferring electrons within the cyclic electron transport pathway of photosynthesis activity;  Iron ion binding; Oxidoreductase activity, acting on diphenols and related substances as donors, oxygen as acceptor; Oxygen evolving activity | 5.30 | 37898.01 | -2.900649325 |
| A0A1B6QEH8 | ATP synthase gamma chain, chloroplastic | Membrane | Proton motive force-driven ATP synthesis | Proton-transporting ATP synthase activity, Rotational mechanism | 7.52 | 39695.94 | -2.896679206 |
| C5Z4V0 | Peptidase S9 prolyl oligopeptidase catalytic domain-containing protein | Membrane | Proteolysis | Serine-type peptidase activity | 8.75 | 54425.97 | -2.994401341 |
| A1E9R0 | Photosystem II CP43 reaction center protein | Plastid, chloroplast thylakoid membrane | Photosynthetic electron transport in photosystem II | Chlorophyll binding; Electron transporter, transferring electrons within the cyclic electron transport pathway of photosynthesis activity;  Metal ion binding | 6.34 | 50098.80 | -2.893746722 |
| A0A1W0W7T6 | 4-alpha-glucanotransferase | Cytosol | Maltose catabolic process | 4-alpha-glucanotransferase activity;  Beta-maltose 4-alpha-glucanotransferase activity; Heteropolysaccharide binding;  starch binding | 5.89 | 108225.76 | -2.873370003 |
| A0A1B6QQR5 | Glyceraldehyde-3-phosphate dehydrogenase | None predicted | Glucose metabolic process | Glyceraldehyde-3-phosphate dehydrogenase (NAD+) (phosphorylating) activity; NAD binding; NADP binding | 5.88 | 46763.02 | -2.872291604 |
| C5X956 | FAD-binding domain-containing protein | None predicted | None predicted | FAD binding;  oxidoreductase activity | 9.50 | 47487.82 | -2.806989276 |
| A0A1B6QD19 | Glycosyl hydrolase family 13 catalytic domain-containing protein | Chloroplast starch grain | Starch catabolic process | Isoamylase activity | 6.28 | 86855.97 | -2.788109712 |
| C5X4E7 | Uncharacterized protein | Endosome;  Lytic vacuole | SNARE complex assembly | SNARE binding | 5.58 | 37689.75 | -2.76783475 |
| A0A1Z5RF98 | Peptidase M48 domain-containing protein | Membrane | Proteolysis | ATP binding; ATP-dependent peptidase activity;  Metalloendopeptidase activity | 9.08 | 33815.04 | -2.761966056 |
| C5XX72 | 4-hydroxy-3-methylbut-2-en-1-yl diphosphate synthase | Chloroplast | Isopentenyl diphosphate biosynthetic process, methylerythritol 4-phosphate pathway; Response to bacterium; Systemic acquired resistance, salicylic acid mediated signaling pathway; Terpenoid biosynthetic process | 4 iron, 4 sulfur cluster binding;  4-hydroxy-3-methylbut-2-en-1-yl diphosphate synthase activity;  Iron ion binding | 5.55 | 82069.07 | -2.76094902 |
| C5XPV2 | Alpha-1,4 glucan phosphorylase | Cytoplasm | Glycogen catabolic process | Glycogen phosphorylase activity; Linear malto-oligosaccharide phosphorylase activity; Pyridoxal phosphate binding; SHG alpha-glucan phosphorylase activity | 7.04 | 94479.35 | -2.7753994145 |

**NB:** Fold change values are shown for salinity where + and − symbols depict up and downregulation of the identified proteins, respectively.

**Appendix C**

**Table 3:** List of differentially expressed dual (15% PEG + 200 mM NaCl) responsive proteins compared to the control identified by LC-MS-MS in treated *P. glaucum* (Information obtained from UniProtKB, Expasy, and WoLF PSORT databases).

| Accession number | Protein name | Subcellular location | Biological processes | Molecular function | Pi | Mr | Fold change (AVG Log 2 Ratios) |
| --- | --- | --- | --- | --- | --- | --- | --- |
| A1E9V5 | Cytochrome b6-f complex subunit 4 | Plastid, chloroplast thylakoid membrane | Mitochondrial electron transport, ubiquinol to cytochrome c; Photosynthetic electron transport chain; Proton transmembrane transport | Electron transporter, transferring electrons within cytochrome b6/f complex of photosystem II activity; Electron transporter, transferring electrons within the cyclic electron transport pathway of photosynthesis activity | 6.56 | 17505.52 | -4.38033 |
| C5YV95 | Sugar phosphate transporter domain-containing protein | Plastid, chloroplast membrane | Monocarboxylic acid transport | Antiporter activity; Carboxylic acid transmembrane transporter activity; Organophosphate ester transmembrane transporter activity | 9.86 | 45116.04 | -4.17254 |
| C5YNV7 | J domain-containing protein | Chloroplast thylakoid membrane; Endoplasmic reticulum | None predicted | None predicted | 4.95 | 25018.65 | -3.78187 |
| A4ZVI4 | Starch synthase, chloroplastic/amyloplastic | Plastid, chloroplast; Plastid, amyloplast | Starch biosynthetic process | Glycogen (starch) synthase activity | 6.23 | 66463.62 | -3.71043 |
| A0A1Z5RFS3 | RCK N-terminal domain-containing protein | Plastid, chloroplast envelope | None predicted | Potassium: proton antiporter activity | 5.28 | 124531.47 | -3.54951 |
| C5YLV3 | adenylate kinase | Chloroplast; Cytoplasm | Energy homeostasis; Nucleoside triphosphate biosynthetic process;  Phosphorylation | Adenylate kinase activity;  ATP binding;  Cytidylate kinase activity;  Nucleoside diphosphate kinase activity | 7.72 | 31041.29 | -3.32216 |
| C5YT06 | VOC domain-containing protein | None predicted | None predicted | None predicted | 6.32 | 17718.13 | -3.25197 |
| C5YMN8 | Uncharacterized protein | Membrane | Malate transmembrane transport; Mitochondrial transport;  Organophosphate ester transport;  Oxaloacetate transport; Phosphate ion transmembrane transport;  Succinate transmembrane transport; Sulfate transport; thiosulfate transport | antiporter activity;  malate transmembrane transporter activity;  oxaloacetate transmembrane transporter activity;  succinate transmembrane transporter activity;  sulfate transmembrane transporter activity;  thiosulfate transmembrane transporter activity | 10.21 | 34341.12 | -3.19166 |
| A1E9V4;C5XL99 | Cytochrome b6; Cytochrome b/b6 N-terminal region profile domain-containing protein | Plastid, chloroplast thylakoid membrane; Membrane | Photosynthesis; Respiratory electron transport chain | Electron transporter, transferring electrons within cytochrome b6/f complex of photosystem II activity;  Metal ion binding;  Electron transfer activity | 8.89; 9.06 | 24164.57; 25946.55 | -3.14672 |
| A0A1B6QII6 | Uncharacterized protein | None predicted | None predicted | None predicted | 5.20 | 19465.76 | -3.09076 |
| C5XBU3 | peptidylprolyl isomerase | None predicted | None predicted | peptidyl-prolyl cis-trans isomerase activity | 10.06 | 23044.29 | -3.08318 |
| A0A1Z5RCK4 | 1,4-alpha-glucan branching enzyme | Plastid, amyloplast | Glycogen biosynthetic process; Starch biosynthetic process;  Starch metabolic process | 1,4-alpha-glucan branching enzyme activity; 1,4-alpha-glucan branching enzyme activity (using a glucosylated glycogenin as primer for glycogen synthesis); Cation binding;  Hydrolase activity, hydrolyzing O-glycosyl compounds | 5.30 | 93101.24 | -3.0762 |
| A0A194YU41 | Cytochrome P450 | None predicted | Xanthophyll biosynthetic process | Carotene beta-ring hydroxylase activity; Heme binding;  Iron ion binding; Oxidoreductase activity, acting on paired donors, with incorporation or reduction of molecular oxygen | 5.93 | 70015.00 | -3.01835 |
| C5Z5R1 | Fructose-bisphosphate aldolase | Cytosol | Fructose 1,6-bisphosphate metabolic process; Glycolytic process | Fructose-bisphosphate aldolase activity | 7.57 | 37985.83 | -2.93361 |
| A0A1Z5RF98 | Peptidase M48 domain-containing protein | Membrane | Proteolysis | ATP binding; ATP-dependent peptidase activity;  Metalloendopeptidase activity | 9.08 | 33815.04 | -2.90151 |
| A0A1B6QD19 | Glycosyl hydrolase family 13 catalytic domain-containing protein | Chloroplast starch grain | Starch catabolic process | Isoamylase activity | 6.28 | 86855.97 | -2.89379 |
| C5YJS0 | RRM domain-containing protein | Cytoplasm;  Nucleus;  Ribonucleoprotein complex | None predicted | mRNA binding | 9.57 | 28788.87 | -2.83957 |
| A0A194YQ92 | CBM20 domain-containing protein | Chloroplast | Protein autophosphorylation;  Starch metabolic process | ATP binding;  carbohydrate kinase activity; Phosphoglucan, water dikinase activity;  Starch binding | 5.55 | 133466.71 | -2.7904 |
| C5XZR3 | Peptidase A1 domain-containing protein | None predicted | Proteolysis | Aspartic-type endopeptidase activity | 7.94 | 45299.07 | -2.73092 |
| A1E9V0 | Photosystem II CP47 reaction center protein | Plastid, chloroplast thylakoid membrane | Photosynthetic electron transport in photosystem II | Chlorophyll binding; Electron transporter, transferring electrons within the cyclic electron transport pathway of photosynthesis activity | 6.06 | 5607034 | -2.67077 |
| A0A1Z5RNX3 | Uncharacterized protein | None predicted | None predicted | None predicted | 10.24 | 16202.60 | -2.66339 |
| A0A1B6P7G2 | AB hydrolase-1 domain-containing protein | None predicted | None predicted | Catalytic activity | 8.22 | 51816.83 | -2.65408 |
| K22E_HUMAN | Keratin, type II cytoskeletal 2 epidermal | Cytoplasm | Epidermis development;  Intermediate filament organization;  Keratinization; Keratinocyte activation; Keratinocyte development; Keratinocyte migration;  Keratinocyte proliferation; Peptide cross-linking; positive regulation of epidermis development | Cytoskeletal protein binding;  Structural constituent of cytoskeleton;  Structural constituent of skin epidermis | 8.07 | 65393.22 | -2.62236 |
| A0A1W0VR99 | DUF1995 domain-containing protein | None predicted | None predicted | None predicted | 6.98 | 43467.30 | -2.59082 |
| A0A1W0VRZ3 | FAD/NAD(P)-binding domain-containing protein | None predicted | Aerobic electron transport chain;  Phylloquinone biosynthetic process | Hydrolase activity;  NAD(P)H dehydrogenase (quinone) activity | 5.59 | 92394.16 | -2.58536 |
| C5Y039 | Lactoylglutathione lyase | Cytoplasm | Methylglyoxal catabolic process to D-lactate via S-lactoyl-glutathione | Lactoylglutathione lyase activity;  Metal ion binding | 5.84 | 37769.46 | -2.56368 |
| A1E9Q9 | Photosystem II D2 protein | Plastid, chloroplast thylakoid membrane | Photosynthetic electron transport in photosystem II | Chlorophyll binding; electron transporter, Transferring electrons within the cyclic electron transport pathway of photosynthesis activity; Iron ion binding;  Oxygen evolving activity | 5.34 | 39402.79 | -2.5608 |
| A1E9R0 | Photosystem II CP43 reaction center protein | Plastid, chloroplast thylakoid membrane | Photosynthetic electron transport in photosystem II | Chlorophyll binding; Electron transporter, Transferring electrons within the cyclic electron transport pathway of photosynthesis activity;  Metal ion binding | 6.34 | 50098.80 | -2.56028 |
| C5Y1S1 | J domain-containing protein | Endoplasmic reticulum | None predicted | None predicted | 6.54 | 27262.85 | -2.55697 |
| A0A1B6QCQ7 | Chlorophyll a-b binding protein, chloroplastic | Plastid, chloroplast thylakoid membrane | Photosynthesis, light harvesting in photosystem I;  Response to light stimulus | Chlorophyll binding | 8.32 | 30059.03 | -2.55217 |
| A1E9S3 | Photosystem I P700 chlorophyll a apoprotein A2 | Plastid, chloroplast thylakoid membrane | Photosynthesis | 4 iron, 4 sulfur cluster binding;  Chlorophyll binding;  Electron transfer activity;  Magnesium ion binding | 6.63 | 82517.87 | -2.5015 |
| A0A1Z5RD75 | Glycine--tRNA ligase | Cytoplasm;  Mitochondrion | Mitochondrial glycyl-tRNA aminoacylation | ATP binding;  Glycine-tRNA ligase activity | 5.97 | 76345.04 | -2.54251 |
| K2C1_HUMAN | Keratin, type II cytoskeletal 1 | None predicted | None predicted | None predicted | 8.15 | 65867.96 | -2.44164 |
| C5YT85 | Thioredoxin domain-containing protein | Chloroplast;  Cytoplasm | Hydrogen peroxide catabolic process; Plastid organization | Oxidoreductase activity, acting on a sulfur group of donors, disulfide as acceptor; protein-disulfide reductase activity | 8.69 | 19333.96 | -2.37902 |
| C5X4E7 | Uncharacterized protein | Endosome;  lytic vacuole | SNARE complex assembly | SNARE binding | 5.58 | 37689.75 | -2.33726 |
| A0A194YHK2;A0A1W0VU28 | H(+)-exporting diphosphatase | Membrane | None predicted | Inorganic diphosphate phosphatase activity;  Pyrophosphate hydrolysis-driven proton transmembrane transporter activity | 5.18;5.26 | 79704.36;83911.69 | -2.33213 |
| C5YEE1 | VDE lipocalin domain-containing protein | Membrane | Chlorophyll metabolic process;  Xanthophyll cycle | Violaxanthin de-epoxidase activity | 5.26 | 50602.07 | -2.31268 |
| C5WRY9 | Outer envelope pore protein 24, chloroplastic | Plastid, chloroplast outer membrane; Plastid, etioplast membrane | Regulation of monoatomic ion transmembrane transport | Porin activity;  Voltage-gated monoatomic cation channel activity | 9.15 | 24163.36 | -2.29409 |
| C5Y669 | Uncharacterized protein | None predicted | None predicted | None predicted | 6.13 | 39888.15 | -2.28312 |
| C5Y4K7 | DUF3700 domain-containing protein | None predicted | None predicted | None predicted | 5.13 | 25181.75 | -2.2777 |
| A0A1B6PBC1 | 4-coumarate--CoA ligase | Endoplasmic reticulum | Phenylpropanoid metabolic process | 4-coumarate-CoA ligase activity;  Long-chain fatty acid-CoA ligase activity;  Trans-cinnamate-CoA ligase activity | 6.70 | 76582.78 | -2.25589 |
| C5YS19 | MPBQ/MBSQ family SAM-binding methyltransferase profile domain-containing protein | Membrane | Methylation | 2-methyl-6-phytyl-1,4-benzoquinone methyltransferase activity | 9.51 | 38637.12 | -2.24877 |
| A0A194YL80 | Uncharacterized protein | Photosystem II oxygen evolving complex | Photosystem II assembly;  Photosystem II stabilization | Oxygen evolving activity | 5.89 | 34805.81 | -2.2483 |
| A0A1B6QEH8 | ATP synthase gamma chain, chloroplastic | Membrane | Proton motive force-driven ATP synthesis | Proton-transporting ATP synthase activity, rotational mechanism | 7.52 | 39695.94 | -2.23697 |
| C5XWW2 | Uncharacterized protein | VCP-NPL4-UFD1 AAA ATPase complex | ER-associated misfolded protein catabolic process; Ubiquitin-dependent ERAD pathway | Polyubiquitin modification-dependent protein binding | 5.76 | 34591.23 | -2.22731 |
| A0A1W0W7T6 | 4-alpha-glucanotransferase | Cytosol | Maltose catabolic process | 4-alpha-glucanotransferase activity;  Beta-maltose 4-alpha-glucanotransferase activity;  Heteropolysaccharide binding;  Starch binding | 5.89 | 108225.76 | -2.21783 |
| C5XQK1 | Uncharacterized protein | None predicted | None predicted | None predicted | 9.54 | 19253.23 | -2.21575 |

**NB:** Fold change values are shown for dual stress where + and − symbols depict up and downregulation of the ident
